# A phenomics-based approach for the detection and interpretation of shared genetic influences on 29 biochemical indices in southern Chinese men

**DOI:** 10.1186/s12864-019-6363-0

**Published:** 2019-12-16

**Authors:** Yanling Hu, Aihua Tan, Lei Yu, Chenyang Hou, Haofa Kuang, Qunying Wu, Jinghan Su, Qingniao Zhou, Yuanyuan Zhu, Chenqi Zhang, Wei Wei, Lianfeng Li, Weidong Li, Yuanjie Huang, Hongli Huang, Xing Xie, Tingxi Lu, Haiying Zhang, Xiaobo Yang, Yong Gao, Tianyu Li, Yonghua Jiang, Zengnan Mo

**Affiliations:** 1 Center for Genomic and Personalized Medicine, Guangxi Medical University, Nanning, 530021 Guangxi China; 2 Life Sciences Institute, Guangxi Medical University, Nanning, 530021 Guangxi China; 3 Department of chemotherapy, The Affiliated Tumor Hospital of Guangxi Medical University, Nanning, 530021 Guangxi China; 4 Department of Information and Management, Guangxi Medical University, Nanning, 530021 Guangxi China; 5 Department of Biochemistry and Molecular Biology, School of Pre-Clinical Medicine, Guangxi Medical University, Nanning, 530021 Guangxi China

**Keywords:** Phenomics, FAMHES cohort, Biochemical indices, Shared genetics, Lipid metabolism

## Abstract

**Background:**

Phenomics provides new technologies and platforms as a systematic phenome-genome approach. However, few studies have reported on the systematic mining of shared genetics among clinical biochemical indices based on phenomics methods, especially in China. This study aimed to apply phenomics to systematically explore shared genetics among 29 biochemical indices based on the Fangchenggang Area Male Health and Examination Survey cohort.

**Result:**

A total of 1999 subjects with 29 biochemical indices and 709,211 single nucleotide polymorphisms (SNPs) were subjected to phenomics analysis. Three bioinformatics methods, namely, Pearson’s test, Jaccard’s index, and linkage disequilibrium score regression, were used. The results showed that 29 biochemical indices were from a network. IgA, IgG, IgE, IgM, HCY, AFP and B12 were in the central community of 29 biochemical indices. Key genes and loci associated with metabolism traits were further identified, and shared genetics analysis showed that 29 SNPs (*P* < 10^− 4^) were associated with three or more traits. After integrating the SNPs related to two or more traits with the GWAS catalogue, 31 SNPs were found to be associated with several diseases (*P* < 10^− 8^). Using *ALDH2* as an example to preliminarily explore its biological function, we also confirmed that the rs671 (ALDH2) polymorphism affected multiple traits of osteogenesis and adipogenesis differentiation in 3 T3-L1 preadipocytes.

**Conclusion:**

All these findings indicated a network of shared genetics and 29 biochemical indices, which will help fully understand the genetics participating in biochemical metabolism.

## Background

Complex traits are the product of various biological signals and some intermediate traits may be affected either directly or indirectly by these signals [1]. A phenome is the sum of many phenotypic characteristics (phenomics traits) that signifies the expression of the whole genome, proteome and metabolome under a specific environmental influence [2, 3]. The study of phenomes (called phenomics) provides a suite of new technologies and platforms that have enabled a transition from focused phenotype-genotype studies to a systematic phenome-genome approach [4]. Many recent studies have found that, compared to considering only binary patients vs. healthy controls, mapping intermediate steps in disease processes, such as various disease-related clinical quantitative traits or gene expression, is more informative [5, 6].

Pleiotropy, which is a DNA variant or mutation that can affect multiple traits, is a common phenomenon in genetics [7]. For example, Joseph Pickrell and colleagues [8] performed genome-wide association studies (GWAS) of 42 traits or diseases to compare the genetic variants associated with multiple phenotypes and identified 341 loci associated with multiple traits. Heid IM et al [9] performed a GWAS of fasting insulin, high-density lipoprotein cholesterol (HDL-C) and triglyceride (TG) levels to identify 53 loci associated with a limited capacity to store fat in a healthy way, and this multi-trait approach could increase the power to gain insights into an otherwise difficult-to-grasp phenotype. Furthermore, many studies have found that diseases or clinically quantitative traits can be interconnected. For example, increasing circulating fatty acids (Fas) could lead to the development of obesity-associated metabolic complications, such as insulin resistance [10]. Goh et al [11] found that essential human genes tended to encode hub proteins and were widely expressed in multiple tissues. Many shared genetic variants are identified in linkage disequilibrium with variants associated with other human traits or diseases, and these pleiotropic connections connect the human traits together [8, 12]. Therefore, understanding the complex relationships among human traits and diseases is important for learning about the molecular function of hub genes.

The Fangchenggang Area Male Health and Examination (FAMHES) cohort was initiated in 2009 in Fangchenggang City, Guangxi, China. It is a comprehensive demographic and health survey that focuses on investigating the interaction between the environment and genetic factors on men’s health. In a previous study, we reported that biochemical indices are closely associated with disease. For example, higher complement 3 (C3) and complement 4 (C4) were associated with an increase in metabolic syndrome (MetS) [13]. Low serum osteocalcin levels were a potential marker for MetS [14] and impaired glucose tolerance [15]. Uric acid (UA) was positively correlated with the prevalence of MetS [16]. Additionally, a genome-wide assay indicated that genes or loci associated with lipid traits are related to biochemical indices. For example, alcohol consumption and *the ALDH2* rs671 polymorphism affected serum TG levels [17]. Although the role of genetic factors and gene polymorphisms in biochemical indices has been reported, the network of biochemical indices themselves, biochemical indices and genetic types are still puzzling. With the rapid advances in bioinformatics techniques, clarifying the biochemical indices network with genetic types becomes feasible.

The aim of this study was to identify the shared genetics responsible for 29 biochemical indices in the FAMHES cohort using a phenomics approach. Our findings shed light on the relationships between these 29 biochemical indices, including their shared genetic basis and genetic risk loci.

## Results

### Genetic and trait-based characteristics of 1999 samples

**Fig. 1:**
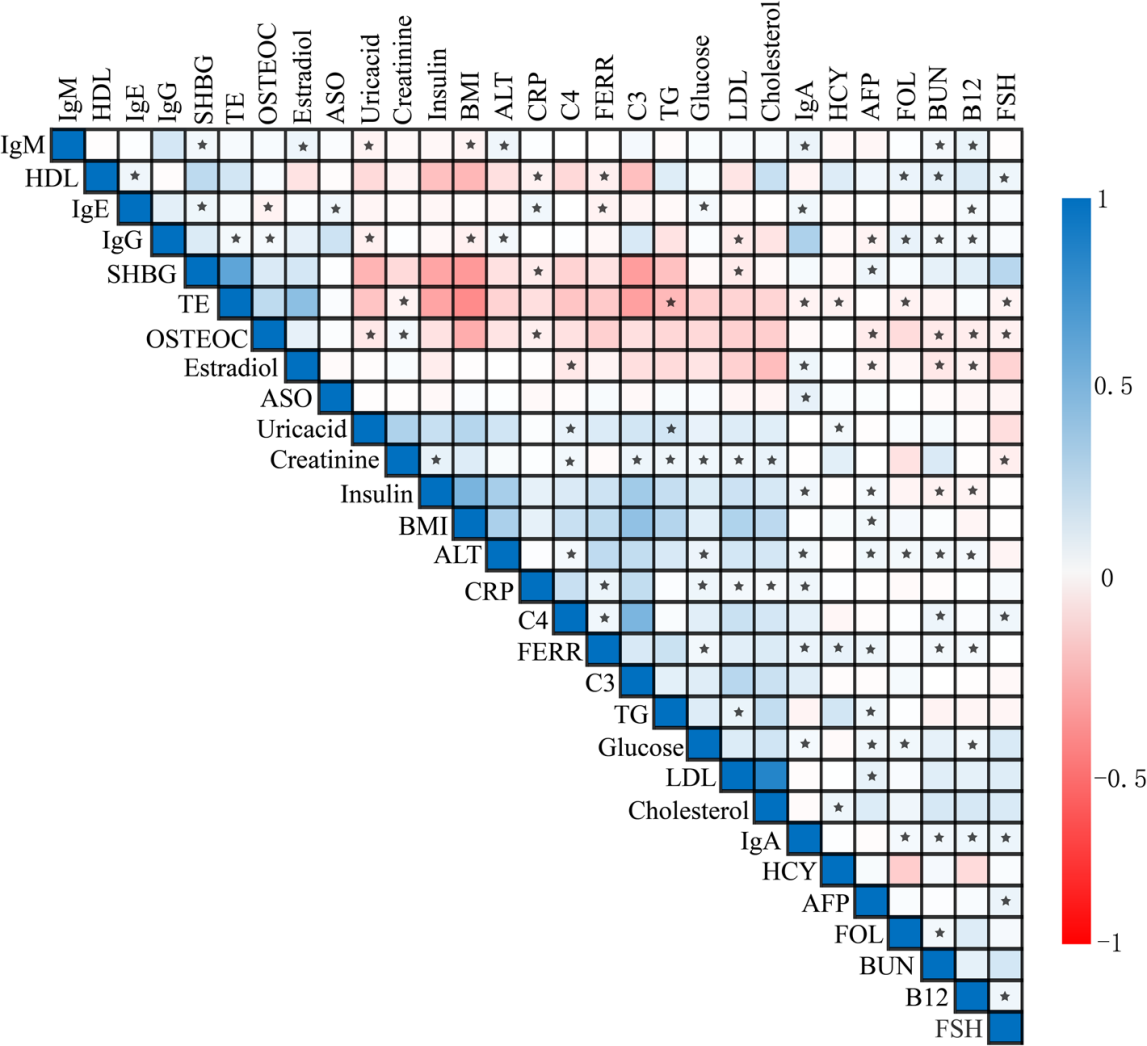
The heatmaps based on the Pearson correlation for 29 biochemical indices in the FAMHES cohort. The coefficient in each cell ranges from − 1 to 1. A negative value denotes a negative correlation, a positive value denotes a positive correlation, 1 indicates a complete correlation, and 0 indicates no correlation. The correlations between clinical quantitative traits shown in this matrix are shown in blue and red. Blue represents a positive correlation, and the darker the colour, the stronger the positive correlation. Red indicates a negative correlation, and the darker the colour, the stronger the negative correlation. If the correlation coefficients were greater than 0.3 or less than − 0.3 and *P* value< 0.01, we considered the pairs to be correlated

A total of 1999 subjects with 29 biochemical indices that passed the QC call rate of 95% were analysed, and a total of 709,211 SNPs in these subjects were subjected to the subsequent genetic analysis. The average GWAS inflation factor for all 29 biochemical indices was 1.029 (range: 0.975–1.060), suggesting that the stratification correlation worked well (Additional file 5: Table S1). The heatmaps based on the Pearson correlation coefficient showed that 106 correlated pairs were found among these 29 traits (correlation coefficient was over 0.3 or less than − 0.3 and the *P* value was less than 0.01) (Fig. 1). In addition, cluster analysis with the hclust package in the R package classified these 29 biochemical indices into 2 groups, with one group including blood urea nitrogen (BUN), cholesterol, glucose, testosterone (TE), follicle-stimulating hormone (FSH), insulin, immunoglobulin G (IgG), homocysteine (HCY), folate (FOL), alpha-fetoprotein (AFP), immunoglobulin A (IgA), low-density lipoprotein cholesterol (LDL-C), immunoglobulin M (IgM), C3, how-density lipoprotein cholesterol (HDL), TGs, and C-reactive protein (CRP). The other group included vitamin B12 (B12), ferritin (FRRR), uric acid, immunoglobulin E (IgE), anti-streptococcus haemolysin “O” (ASO), creatinine, osteocalcin (OSTEOC), oestradiol, sex hormone binding globulin (SHBG), and alanine transaminase (ALT) (Additional file 1: Figure S1). Each group contained common lipid metabolism indices, suggesting that these traits were correlated with lipid metabolism.

### Correlation analysis based on network medicine

For each trait, we used a linear mixed model estimate fixed value, adjusted with PC1 and PC2 of population stratification and age, respectively, to perform a GWAS. A total of 86,556 SNPs (*P* value 1 × 10^− 3^) associated with all 29 biochemical indices were obtained and then annotated using the SNP function database with default parameters and the south Asian population option [18]. A total of 12,521 genes were obtained, and protein-protein interactions were determined using the BioGRID database [19]. A total of 5313 genes with known proteins were obtained, and the interactional network was built with Cytoscape 3 [20]. The topological coefficient, clustering coefficient and degree distribution were important indices to evaluate network nodes. Details of these three factors for 5313 genes are shown in Additional file 2: Figure S2 (A, B, C, D).

**Fig. 2:**
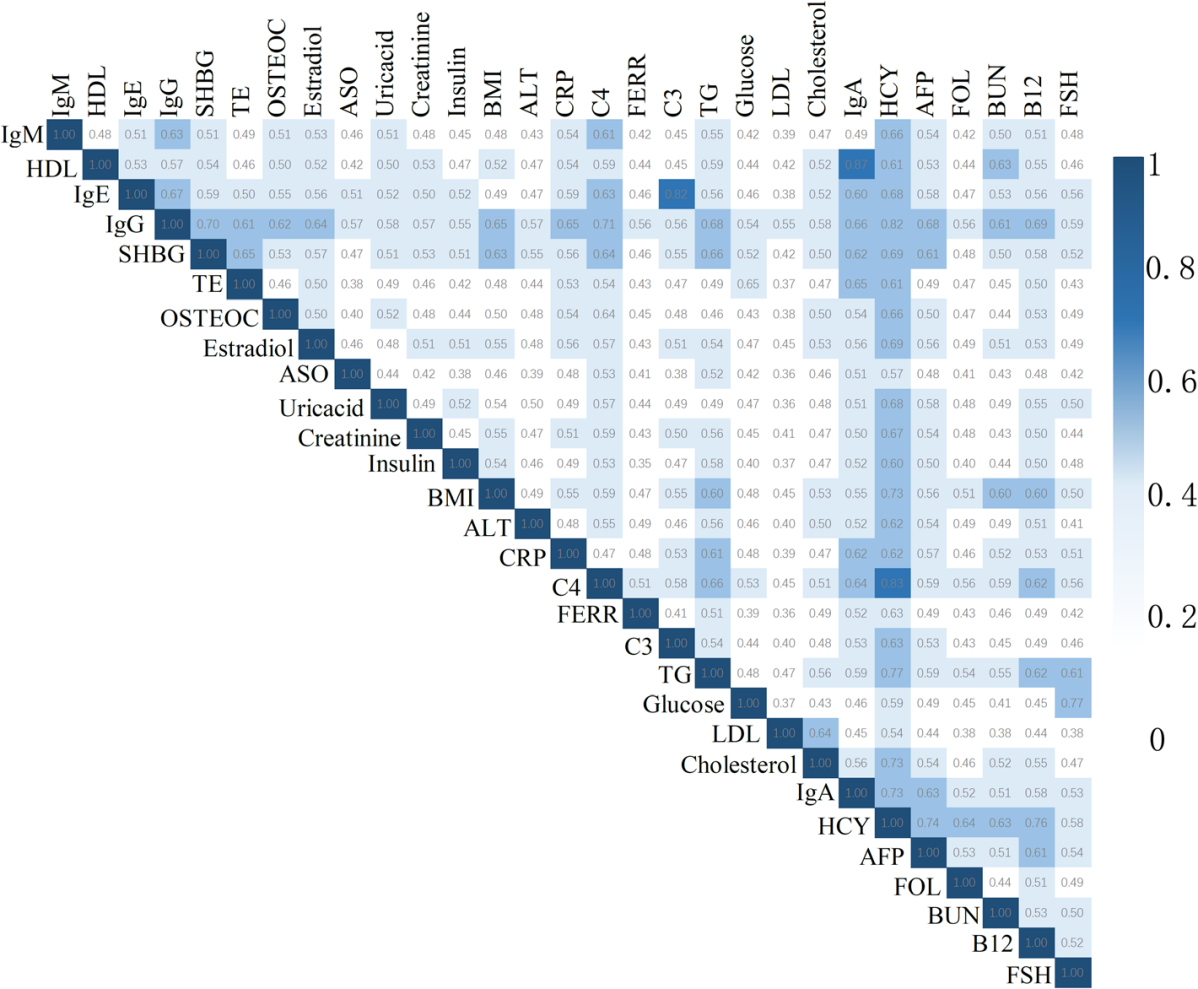
Molecular comorbidity index (MCI) for 29 biochemical indices in the FAMHES cohort. The MCI value is between 0 and 1. The darker blue indicates a stronger correlation between the two clinical biochemical indicators. If the MCI was over 0.6, we considered the pairs to be correlated

The Jaccard correlation matrix heatmaps showed that there were 63 correlated pairs among 435 pairwise combinations among these 29 traits indices with an MCI over 0.6 (Fig. 2). In these pairs, HCY, IgG, SHBG, B12, IgA and C4 were closely related with more than six other traits. However, because the information regarding gene/protein interactions in public databases is limited, interaction information for most of the genes/proteins in this study could not be obtained, and the Jaccard index was computed based on a small number of genes/proteins.

### Correlation analysis based on linkage disequilibrium score regression (LDSC)

**Fig. 3:**
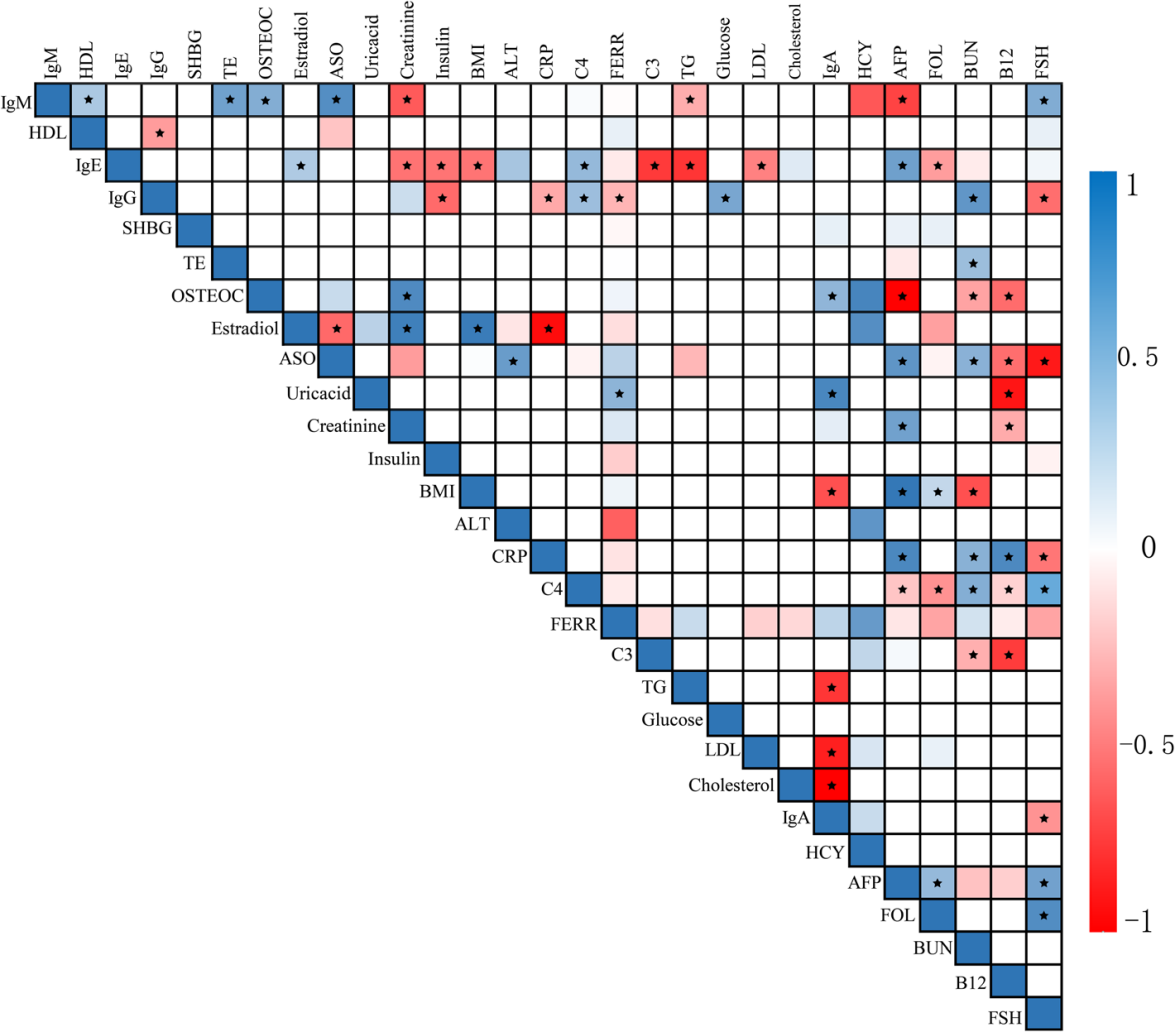
Correlation analysis based on linkage disequilibrium score regression (LDSC) for 29 biochemical indices in the FAMHES cohort. The genetic correlation estimate (Rg) ranges between − 1 and 1. A negative value denotes a negative correlation, a positive value denotes a positive correlation, 1 indicates a complete correlation, and 0 indicates no correlation. The correlations between clinical biochemical indicators shown in this matrix are represented by blue and red. Blue represents a positive correlation, and the darker the colour, the stronger the positive correlation. Red indicates a negative correlation, and the darker the colour, the stronger the negative correlation

Genetics can help to elucidate cause and effect. However, single variants tend to have minor effects, and reverse causation involves an even smaller list of confounding factors. Therefore, interrogating genetic overlap via GWAS that focuses on genome-wide significant SNPs is predicted to be an effective means of mining the correlation between different phenotypes. The GWAS effect size estimate for a given SNP will capture information about SNPs near the linkage disequilibrium [21]. The correlations based on GWAS of the 29 quantitative clinical traits were estimated using cross-trait LDSC. The genetic correlation estimates for all 435 pairwise combinations among these 29 traits. After removing the outlier values, 68 significantly correlated pairs (*p* < 0.05) were found (Fig. 3). The details for these 68 selected pairs of traits are shown in Additional file 6: Table S2.

### Integration and interpretation of important pairs identified by these three methods

To identify the correlation pairs among these three methods, we integrated the correlated traits fitting at least one of the following: Pearson coefficient was greater than 0.3 or less than − 0.3 and *P* value less than 0.01, Jaccard coefficient was greater than 0.6, or P value of LDSC was less than 0.05. In total, 208 correlated pairs among biochemical indices were found; among them 106, 63, 68 correlated pairs were found by Pearson coefficient, Jaccard coefficient, and LDSC, respectively. Only 1 correlated pair was found by all three methods. Ten correlated pairs, both by Pearson coefficient and LDSC were found, 15 by Pearson and Jaccard coefficient, and 5 by Jaccard coefficient and LDSC. (Additional file 3: Figure S3, A). The related traits were integrated if they fulfilled the following conditions: the Pearson coefficient was greater than 0.3 and *P* value less than 0.01, the Jaccard coefficient was greater than 0.6, or the LDSC *p* value was less than 0.05. Six traits (IgA, IgG, HCY, AFP, IgE and B12) were the first top factors in the network of these 29 traits and were related to more than 20 traits. Additionally, IgM, CRP, C4, BUN, TG, creatinine and FSH were the second top factors and connected with more than 15–20 traits, and OSTEOC, oestradiol, glucose, FOL, TE, SHBG, FERR, BMI, ALT and HDL were the third top traits, which correlated with more than 10 traits (Additional file 3: Figure S3, B).

### Genes and SNPs that are potentially important across multiple traits

We selected SNPs with *P* < 10^− 3^ for each trait, resulting in a total of 60,644 SNPs for all 27 traits. The essential genes have a tendency to be expressed in multiple tissues and are topologically and functionally central [12]. After integrating all 5313 genes and removing the free notes in the total network among 29 biochemical indices, 427 genes (with *P* < 10^− 3^ at least one SNP) were correlated with more than 5 traits. After filtering the genes with SNPs (*P* < 10^− 4^), there were 71 genes correlated with more than or equal to 3 traits, especially aldehyde dehydrogenase 2 family member (*ALDH2*), BRCA1 associated protein (*BRAP*), cadherin 13 (*CDH13*) and CUB and Sushi multiple domains 1 (*CSMD1*), which was related to more than 5 traits. In these 71 genes, 38 genes were found to connect more than 5 other genes in the interactional network annotated from the BioGRID database [19] (Additional file 7: Table S3), which showed that essential genes related to multiple traits were located in the central gene interactional network.

**Fig. 4:**
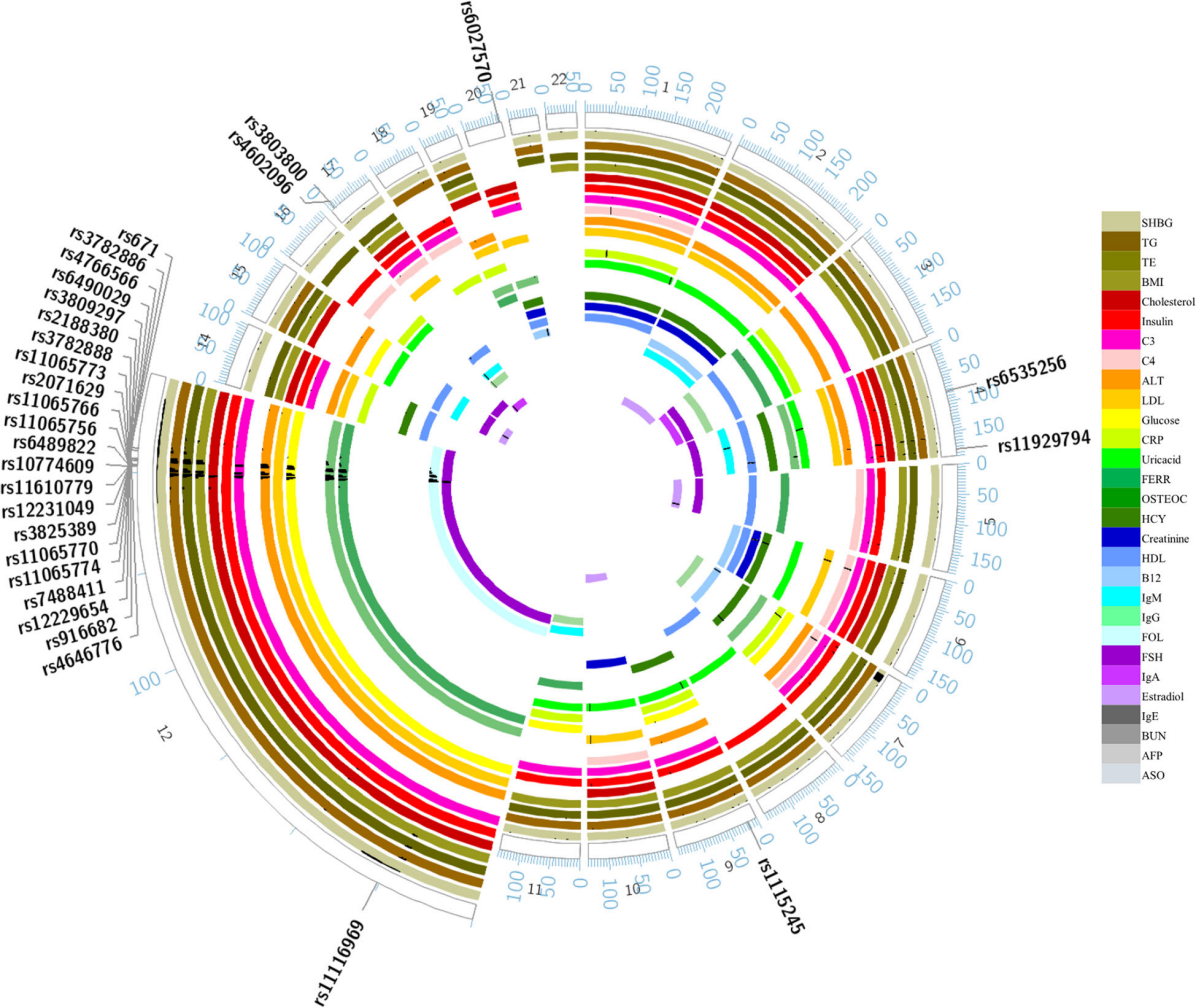
Circos plot of shared SNPs related to more than 3 biochemical indices based on analysis of individuals in the FAMHES cohort. Each plot presents one trait with a specific colour. ASO and IgE have no common SNPs in these 481 SNPs, so they are not in this Circos. The black dash denotes the shared SNPs, and the upper line denotes the significant value with the log (*p* value). The chromosome number is marked on the outside of the Circos plot. The chromosome positions of 29 common sites (*P* value< 10^− 4^) associated with more than four biochemical indices are marked on the outside of the Circos plot

Among all the genome-wide variation SNPs, 481 (*P* < 1✕10^− 3^) were associated with three or more clinical biochemical quantitative traits, and 13 of these 481 SNPs were related to more than 5 traits. In these SNPs, rs12229654 (near cut like homeobox 2 (CUX2)), rs2188380 (located in *CUX2*), rs3809297 (located in *CUX2*) and rs3782886 (located in *BRAP*) were related to more than 10 traits. Six SNPs in *CUX2* were correlated with more than 5 traits, which indicates that *CUX2* should play an important role on this net. In addition, for all the SNPs with *P* < 1 × 10^− 4^, 29 SNPs were related to three or more biochemical indices (Fig. 4). After annotating 29 SNPs with *P* < 1 × 10^− 4^ using the HaploReg database [22], we found that almost all these SNPs were related to enhancer histone binding, promoter DNase binding and transcript binding, which affected protein binding or the presence of eQTLs (Additional file 8: Table S4).

After integrating the SNPs associated with more than 2 traits(*P* < 1 × 10^− 4^) with the GWAS catalogue [23], we found that 31 SNPs in 18 genes were in the GWAS catalogue (Additional file 9: Table S5). Among those SNPs, five SNPs (rs579459, rs649129, rs507666, rs495828, and rs651007) in ABO were associated with more than 10 quantitative traits and diseases. One SNP (rs671) in *ALDH2* was related to 21 traits, six SNPs (rs10519302, rs16964211, rs2305707, rs2414095, rs6493487 and rs727479) in or near CYP19A1 were mainly associated with hormone measurements. This finding supports the idea that shared genetics for traits can produce correlations among these traits.

### The rs671 polymorphism in *ALDH2* affects osteogenic and adipogenic differentiation of 3 T3-L1 preadipocytes

**Fig. 5:**
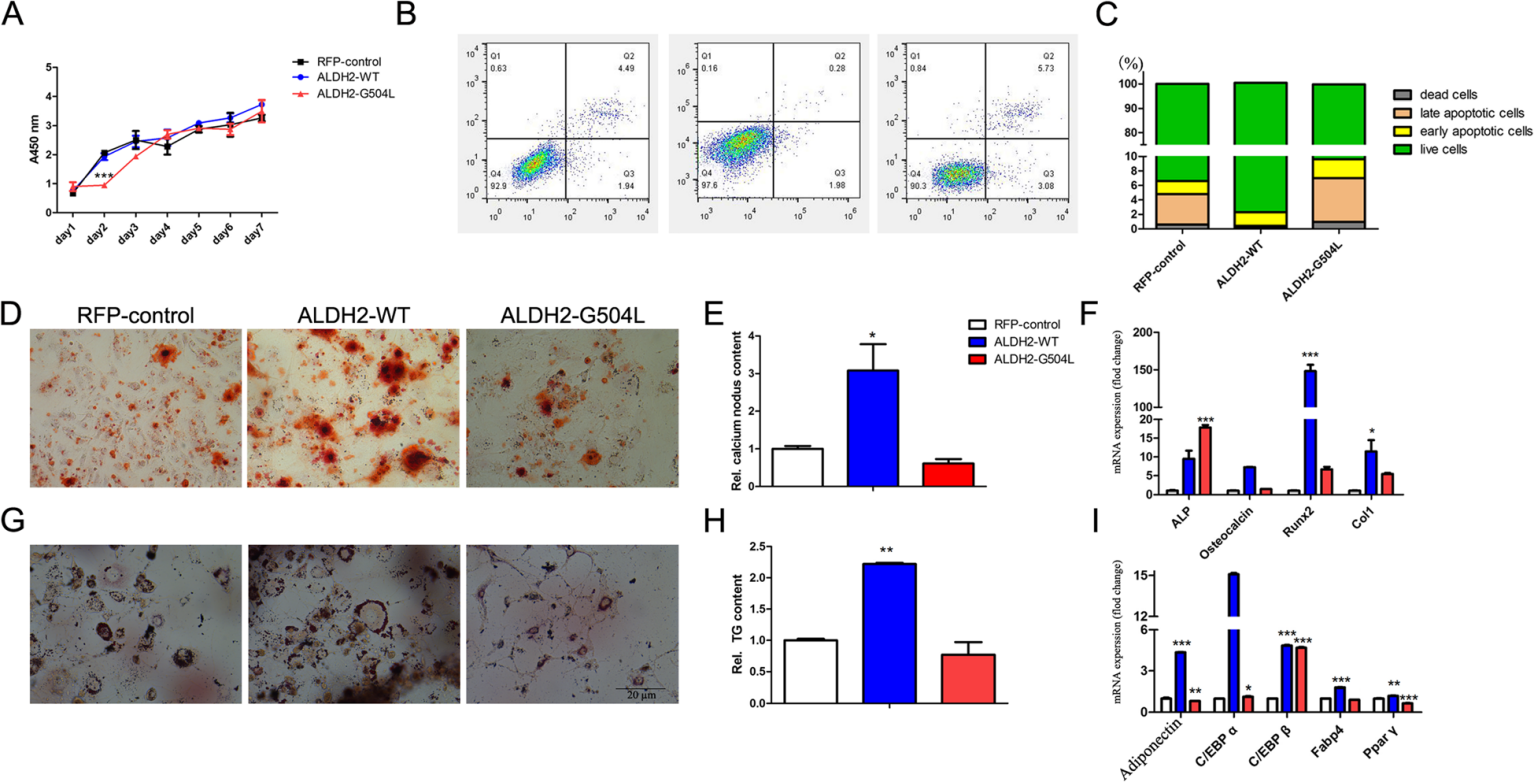
The impact of *ALDH2* rs671 on osteogenic and adipogenic differentiation of 3 T3-L1 preadipocytes. **a** The cell growth curve measured as 450 nm absorbance by using Cell Counting Kit-8 Annexin V-FITC/PI–labelled cells was detected by flow cytometry to measure osteoblast apoptosis. Representative dot plots **b** and quantified data as the percentage of total cells **c** At 7 days after osteoblast induction, cells were stained with Alizarin Red S solution to measure calcium content. Representative photographs **d** and quantified Alizarin red S staining in cells. **e** Expression of osteocalcin-related genes (*AKP*, osteocalcin, *Runx2, Col1*) in ALDH2 WT- or Glu504Lys-overexpressing 3 T3-L1 preadipocytes after 7 days of induction refer to 3 T3-L1 RFP. f At 7 days after adipocyte induction, cells were stained with Oil Red O to measure triglyceride (TG) content. Representative photographs **g** and quantified Oil Red O staining in cells. **h** qPCR analysis of adipogenic (adiponectin, C/EBPα, C/EBPβ, Fabp4, Pparγ) expression in ALDH2 WT- or Glu504Lys-overexpressing 3 T3-L1 preadipocytes after 7 days of induction refer to 3 T3-L1 RFP. **i** Data are shown as the mean ± SE from 3 independent experiments. * *P* < 0.05, **P < 0.01; ***P < 0.001

An interaction between a SNP (rs671) in *ALDH2* was related to 13 traits found in this study. The relationship between rs671 and lipid metabolism or osteocalcin has been found in some studies [24, 25]; however, their function needs to be investigated. Rs671 is a nonsynonymous (ns) SNP (G504 L) in the *ALDH2* gene, which is located on chromosome 12. To evaluate the effects of the rs671 polymorphism on osteogenic and adipogenic differentiation of 3 T3-L1 preadipocytes, a lentivirus vector was used to overexpress *ALDH2*-WT or *ALDH2*-G504 L-mut in 3 T3-L1 preadipocytes (Additional file 4: Figure S4). The cell growth curve of *ALDH2*-G504 L-mut showed no obvious change compared with that of the control, but expression of *ALDH2*-WT induced a significant increase in cell proliferation (Fig. 5a). The cell apoptosis results were consistent with this finding; overexpression of *ALDH2*-WT resulted in a 3.935-fold decrease in late apoptotic cells in comparison to that of *ALDH2*-G504 L-mut or control cells (Fig. 5b, c). We next investigated the impact of the *ALDH2* G504 L mutation on the osteogenic and adipogenic differentiation of 3 T3-L1 preadipocytes. At 7 days after osteoblast induction, cells were subjected to Alizarin red S staining. *ALDH2*-WT cells showed more mineralized nodules than the control cells or those expressing *ALDH2*-G504 L-mut (Fig. 5d, e). In addition, the mRNA expression of osteoblast-related genes, such as alkaline phosphatase (*AKP*), osteocalcin, RUNX family transcription factor 2 (*Runx2*), and collagen type I (*Col1*), was significantly higher in *ALDH2*-WT cells than in *ALDH2*-G504 L-mut or control cells (Fig. 5f). After 7 days of adipogenic induction, the *ALDH2*-WT cells displayed accumulation of lipid vacuoles, as detected by oil red O staining, when compared with *ALDH2*-G504 L-mut or control cells (Fig. 5g, h). The expression levels of adipogenesis-related proteins, such as adiponectin, *C/EBPα* (CCAAT/enhancer binding protein α), *C/EBP*β, adipocyte fatty acid-binding protein (*Fabp4*), and *Pparγ* (peroxisome proliferator-activated receptor), were much higher in *ALDH2*-WT cells than in *ALDH2*-G504 L-mut or control cells (Fig. 5i). Taken together, these results suggest that *ALDH2*-G504 L-mut affected the osteogenic and adipogenic differentiation of 3 T3-L1 preadipocytes.

## Discussion

A network of shared genetics and 29 biochemical indices were found in this research study. Not only did one intermediate phenotype have multiple associated SNPs, interestingly, one SNP associating with multiple intermediate phenotypes was also common. The phenomenon of some genes or loci having the ability to affect multiple distinct phenotypic traits is called pleiotropy. Increasing attention has been paid to pleiotropy. In 2011, according to the data of the NIH GWAS website, Sivakumaran found that nearly 5% of SNPS and 17% of genes or gene regions were related to two or more diseases or traits [26]. In 2018, Chesmore used the same method and database and found that 44% of genes or gene regions were associated with two or more diseases or traits, a nearly two-fold increase to that of Sivakumaran S [27]. It has been suggested that pleiotropy facilitates the accurate diagnosis and treatment of human diseases [28]. Moreover, pleiotropy research is also helpful for understanding the association between sequence variation and phenotype in plants or animals. Gene co-expression networks and novel mutations associated with many phenotypic traits were identified in maize [29, 30]. It has been proven that the wing shape of Drosophila is affected by multiple genetic sites [31].

Immunoglobulin is produced by plasma cells and lymphocytes and is characteristic of these types of cells and plays an essential role in the body’s immune system. In this study, we found that IgG, IgA, IgE and IgM were the central traits in the biochemical indices network, and these traits could be linked to 19 or more traits. HCY, a naturally occurring amino acid found in blood plasma, plays a central role in biochemical indices by connecting with 23 traits. High levels of HCY have been associated with several body dysfunctions, such as vasculature [32] and endothelial injury [33]. Interestingly, vitamin B12 was identified as having a central role in the biochemical indices network by correlating to 21 other traits. Similar to previous studies, vitamin B12 correlates with several quantitative traits, such as bone mineral density, FOL and FERR [34–36].

Pleiotropy refers that some genes or loci that have the ability to affect multiple distinct phenotypic traits. After integrating all the related genes among 29 biochemical indices, surprisingly, *ALDH2* and *BRAP* can be related to 9 traits and are connected with 19 and 13 genes, respectively. *ALDH2* belongs to the aldehyde dehydrogenase family of proteins, which is the second enzyme of the major oxidative pathway of alcohol metabolism. *ALDH2* dysfunction will lead to several diseases, such as cancer [33, 37], alcoholic fatty liver [38], and cardiovascular diseases [39]. *BRAP* is a cytoplasmic protein, which can bind to the nuclear localization signal of BRCA1 and other proteins [40]. The polymorphisms in this gene are associated with myocardial infarction [41] and metabolic syndrome [42]. Additionally, the common *CSMD1* was related to 8 traits. *CSMD1* is a large (~ 390 kDa) membrane-bound complement inhibitor [43]. Mutations of this gene participate in complement activation and inflammation in the central nervous system, which leads to Parkinson’s disease [44]. These three genes may be hub genes in biochemical indices networks.

If the SNPs located in sites related to promoter DNase binding, enhancer histone binding and transcript binding, the marginally significant SNPs play regulatory roles affecting protein binding or the presence of eQTL [45, 46]. In this research, 29 SNPs (*P* < 10–4) were associated with three or more traits and correlated with each other. These results revealed that the shared regulatory genetics are most likely to drive association signals and play important roles in clinical biological function. This phenomenon may provide important “scaffolding” to support a framework to explore the basic mechanism of biochemical indices.

Shared genetics are commonly used to build disease-diseased relationship and mine the common disorder of diseases [47, 48]. An important general insight from this study was that associated genes across traits tend to gather in trait-specific network modules. We found that 31 SNPs in 18 genes were associated with several traits and diseases; five SNPs (rs579459, rs649129, rs507666, rs495828 and rs651007) of ABO were associated with cholesterol and LDL levels. Six SNPs (rs10519302, rs16964211, rs2305707, rs2414095, rs6493487, rs727479) of CYP19A1 were associated with oestradiol levels. Rs671 in ALDH2 was associated with glucose, OSTEOC, and SHBG levels. These findings suggest that shared genetics on traits can produce correlations between different traits of disease. For example, the ABO gene located near 9q34.2 encodes glycosyltransferases related to the first discovered ABO blood group system [49]. The abnormal expression or polymorphism of this gene is correlated with several body dysfunctions, such as ischaemic stroke [50], large artery atherosclerotic stroke [51] and pancreatic cancer [52]. The CYP19A1 gene, located on 15q21.2, encodes a key enzyme for oestrogen biosynthesis. SNPs in CYP19A1 might affect aromatase activity and influence oestradiol levels, thereby impacting human health. Previous research has reported correlations with SNPs of CYP19 and disease, such as polycystic ovarian syndrome [53], coronary heart disease [54], and coronary artery disease (CAD). The ALDH2 gene, located on 12q24.12, encodes aldehyde dehydrogenase, the second enzyme of the major oxidative pathway of alcohol metabolism. Rs671 is nonsynonymous mutation site on exon 12. The rs671 mutation was found to be associated with several traits (BMI, osteocalcin, renal function-related traits [55], response to alcohol consumption [56, 57], triglyceride [17], haematological and biochemical traits [58], intracranial aneurysm [59], mean corpuscular haemoglobin [17]). Using ALDH2 as an example to preliminarily explore its biological function, the in vitro function testing of rs671 played a role in the proliferation and osteogenic and adipogenic differentiation of 3 T3-L1 preadipocytes.

With the emergence of GWAS, a large number of loci and disease-related information were elucidated. However, due to its strict restriction on the *P* value of correlation analysis, a great deal of potential information was lost while significant loci were obtained. Some loci did not achieve a P cut-off value but itself, but if these loci were located in a short range or were involved in similar functions, these lower *p* value loci may also affect biological function [60]. Furthermore, it was challenging to identify common pathways and biological functionality core regulatory networks across loci. During more efficient analysis of these lower p value loci functions, more complex models emerged. Raychaudhuri designed GRAIL to set a lower threshold in considering relatedness for those genes in narrow regions. They systematically examined 370 SNPs from 179 independent loci with *P* < 1 × 10^− 3^, and three gene regions in CD28, PRDM1 and CD2/CD58 were identified that were closely related to rheumatoid arthritis [61]. To assess new asthma risk loci, Demenais interrogated the GWAS catalogue using set P value thresholds from 5 × 10^− 8^ to 10^− 3^, and performed a meta-analysis on genetic variation and blood indexes and environmental exposure histories [62]. Kostem performed a follow-up analysis of SNPs associated with disease by setting a lower cut-off value and then analysed the particular values of the tag SNP statistic, pairwise correlation, and the effect size of the candidate SNP [63].

Because there are no mature methods of research on the genetic relationship between traits at the level of genome-wide summary statistics, we set a lower threshold value for obtaining more SNPs for analysis, and then analysed the association of these candidate SNPs by three different methods: Pearson correlation coefficient, LDSC or Jaccard correlation. As we show, even with three different calculation methods, most of the top important traits are similar. Of these, IgA, IgG, HCY, AFP, IgE and B12 were the first top factors in the network. Our research is an experimental attempt to assess the network of shared genetics and 29 biochemical indices.

## Conclusion

We investigated the correlations among 29 biochemical indices through three biological information methods. First, we found that IgA, IgG, IgE, IgM, HCY, AFP and B12 were in the central community of 29 biochemical indices. Second, the shared genetics analysis showed that 29 SNPs (*P* < 10^− 4^) were associated with more than 3 traits. Thirty-one SNPs were associated with several diseases (P < 10^− 8^) by integrating the SNPs related with 2 or more traits with the GWAS catalogue. Third, using *ALDH2* as an example to preliminarily explore its biological function, we found that the rs671 (*ALDH2*) polymorphism could affect the osteogenic and adipogenic differentiation of 3 T3-L1 preadipocytes. We clarified that 29 biochemical indices were from a network and that hub variations/genes played a vital role in biological processes. These findings highlight a network of shared genetics and 29 biochemical indices.

## Methods

### Study samples

Our study included 2012 unrelated healthy Chinese men aged 20–69 years from the FAMHES [14, 15], which was conducted among non-institutionalized Chinese men in Guangxi and was designed to investigate the effects of environmental and genetic factors and their interaction with the development of age-related chronic diseases. Men aged ≥18 years were requested to participate in the study upon large-scale physical examination at the Medical Center of Fangchenggang First People’s Hospital from September 2009 to December 2009. The included participants all self-reported that they were free of hyperthyroidism, diabetes mellitus, stroke, coronary heart disease, rheumatoid arthritis, impaired hepatic or renal function, and tumours. Our study research protocol was approved by the Guangxi Medical University Ethics Committee. All participants provided written informed consent prior to participation in this study.

### Measurements of 29 biochemical indices

Overnight (≥8 h) fasting venous blood specimens were obtained between 7:00 am and 10:00 am, and serum samples were extracted and stored at − 80 °C. Triglyceride, cholesterol, HDL-C, LDL-C, glucose, ALT, BUN, uric acid and creatinine were measured enzymatically on a Dimension-RxL Chemistry Analyzer (Dade Behring, Newark, DE) in the Department of Clinical Laboratory Science at the Fangchenggang First People’s Hospital. CRP, C3, C4, IgA, IgE, IgG, IgM, and ASO were measured with immunoturbidimetric methods on a HITACHI 7600 Biochemistry Analyzer (Hitachi Corp, Tokyo, Japan). Ferritin, folate and vitamin B12, TE, oestradiol, FSH, SHBG, insulin, AFP and OSTEOC were measured with the same batch of reagents by electrochemiluminescence immunoassay and HCY assayed by enzyme cycle method using a COBAS 6000 system E601 (Elecsys module) Immunoassay Analyzer (Roche Diagnostics, GmbH, Mannheim, Germany).

### SNP genotyping and quality control (QC) analysis

Genome-wide SNP genotyping was performed with an Illumina Omni 1 M chip (Illumina, San Diego, USA). Among 2012 genotyped subjects, 1999 passed the QC call rate of 95% and were included in the final data analysis. A total of 709,211 SNPs in these subjects passed the QC criteria as follows: the *P* value for the Hardy-Weinberg equilibrium (HWE) test was greater than 1 × 10^− 3^, the minor allele frequency (MAF) was greater than 0.01, and the genotype call rate was greater than 95%. The inferred genotypes of SNPs in the genome that were not directly genotyped were computed by the IMPUTE program [64] (e.g., SNPs catalogued in HapMap Phase II CHB population release #24). All genotypes with a posterior probability of > 90% based on IMPUTE software imputation were retained.

### Jaccard coefficient

Phenotypes are linked if they share alterations in genetics. The pathobiology of human diseases might be understood by creating molecular and phenotypic networks [65, 66]. We used the SNP function [18] (https://snpinfo.niehs.nih.gov/) tool to identify the genes containing all of the SNPs for which the P value for the GWAS was less than 1 × 10^− 3^. The human interactome was obtained by combining protein-protein interaction (PPI) information from the BioGRID database [19].

We built correlations among 29 clinical phenomes based on the common genes/proteins between two traits. To minimize the bias in estimating the correlation between two given traits, we calculated the molecular comorbidity index (MCI) by adapting the formula from Grosdidier S [67] to further consider the different coefficients of distance between the two diseases. The MCI was defined as follows:

$$ {MCI}_{trait1, trait2}=\left(\left({proteins}_{trait1}\cap {proteins}_{trait2}\right)\cup {proteins}_{trait1\to trait2}\cup {proteins}_{trait2\to trait1}\right)/\left({proteins}_{trait1}\cup {proteins}_{trait2}\right) $$

Where *proteins*_*trait*1_ and *proteins*_*trait*2_ are the proteins related to clinical traits 1 and 2, respectively. *proteins*_*trait*1 → *trait*2_ are those proteins related to trait 1 that interact with the proteins associated with trait 2 (and vice versa *proteins*_*trait*2 → *trait*1_). The two operators ∩ and ∪ denote the intersection and union between the two sets of elements (*proteins*_*trait*1_ and *proteins*_*trait*2_, respectively).

### Correlation analysis by LDSC

The genetic correlations derived from the summary statistics were evaluated by the GWAS effect size for a given SNP and integrated the effects of all SNPs that were in linkage disequilibrium (LD) with that SNP. The LDSC (which targets genetic correlation) uses variants across the whole genome and is a symmetrical (i.e., nondirectional) analysis for the risk factor and the outcomes [21]. In short, LDSC assumes that, for polygenic traits, SNPs will also capture information about SNPs near the LD. This relationship between the LD and the associated signal can also be used to test the relationship between the two traits for all SNPs in the genome. To further elucidate the correlations of these 29 biochemical indices in FAMHES from the genetic architecture, we applied LDSC to estimate the correlation of these 29 traits.

### Osteogenic and adipogenic differentiation of 3 T3-L1 preadipocytes

Full-length ALDH2-WT and ALDH2-G504 L-mut cDNA were cloned into the pTSBOE-CMV-MSC-3flag-EF1-tRFP-F2A-Puro lentivirus vector (Quanyang, Shanghai). The 3 T3-L1 preadipocytes were cultured in Dulbecco’s modified Eagle’s medium (DMEM) with 10% foetal bovine serum (FBS) at 37 °C in a humidified atmosphere with 5% CO_2_. The osteoblast-inducing medium used was α-MEM (α-minimum Eagle’s medium) containing 10% FBS (foetal bovine serum), 100 nM dexamethasone, 5 mM β-phosphoglyceride and 5 μg/mL vitamin C. The adipogenesis-inducing medium included A and B medium. The A medium was DMEM containing 10% FBS, 100 nM dexamethasone, 0.5 mM 3-isobutyl-1-methylxanthine and 5 μg/mL insulin. The B medium was DMEM containing 10% FBS and 5 μg/mL insulin. For adipocyte induction, cells were cultured for two cycles of A medium for 2 days and then B medium for 1 day. Cell proliferation was measured by a CCK-8 assay according to the manufacturer’s instructions (DOJINDO, Japan). Cell apoptosis was examined by Annexin V-APC/7-AAD staining followed by flow cytometry detection. For Oil Red O or Alizarin Red S staining, cells were fixed with 4% paraformaldehyde for 30 min and stained with 4% Oil Red O solution or 0.4% Alizarin Red S. Lipid droplets and calcium nodules were quantified using ImageJ software. Cellular RNA was extracted using an RNA extraction kit (Promega, China). Reverse transcription was performed with the Transcriptor Reverse Transcriptase Kit (Kangwei, China). Quantitative reverse transcriptase-PCR was performed using a Roche Light Cycler 480 and KANGWEI qPCR Kit (KANGWEI, China). Per-primer sequences are listed in Additional file 10: Table S6.

### Statistical analysis

The correlations among the 29 biochemical indices were computed by the CORR procedure using SAS 9.0 and defined as the Pearson correlation coefficient between the rank variables. With the exception of BUN, HCY, B12, FERR, OSTEOC, creatinine, uric acid, cholesterol, HDL, LDL, TE and C3, 17 traits without normal distribution were logarithmically transformed to normalize the distribution. The association of the SNPs with 29 clinical quantitative traits was evaluated using a linear regression adjusted for population stratification factors (PC1 and PC2) and age. Population stratification was evaluated by a principal component approach with EIGENSTRAT software [68].

### Availability of data and materials

The datasets generated and analysed during the current study are available in the Genome variation Map (GVM) of National Genomics Data Center (NGDC) (Accession Number: GVM000052).

## Supplementary information

**Additional file 1: Fig. S1.** The cluster dendrogram for the 29 biochemical indices from the FAMHES cohort created with the hclust win R package. In this analysis, two main clusters were produced among these 29 traits. FERR (ferritin), CRP (C-reactive protein), C3 (complement 3), C4 (complement 4), AFP (serum alpha-fetoprotein), TG (triglycerides), LDL (low density lipoprotein), ALT (alanine transaminase), BMI (body mass index), ASO (anti streptolysin) (anti-streptolysin “O”), IgG (immunoglobulin G), IgA (immunoglobulin A), IgM (immunoglobulin M), BUN (blood urea nitrogen), FSH (follicle-stimulating hormone), HDL (high-density lipoprotein), TE (testosterone), SHBG (sex hormone binding globulin), IgE (immunoglobulin E), B12 (vitamin B12), HCY (homocysteine).

**Additional file 2: Fig. S2.** Network characteristics of 5313 associated genes for 29 biochemical indices in individuals from the FAMHES cohort were analysed by Cytoscape. (A) Topological coefficient, (B) degree, (C) clustering coefficient, and (D) closeness centrality.

**Additional file 3: Fig. S3.** The integration of correlated traits from three methods. (A) Venn diagram of the integration of correlated traits from three methods. (B) The related traits were integrated if they fulfilled the following conditions: the Pearson coefficient was greater than 0.3, the *P* value was less than 0.01, the Jaccard coefficient was greater than 0.6, or the LDSC *p* value was less than 0.05. Each testing method was denoted by a specific colour: green for Jaccard, and blue for LDSC.

**Additional file 4: Fig. S4.** A lentiviral vector was used to overexpress ALDH2-WT or ALDH2-G504 L-mut in 3 T3-L1 preadipocytes. (A) Localization of the Glu504Lys substitution mutation in *ALDH2*. Ex: exon. (B) The plasmid used to express the ALDH2-Gluc504Lys mutant protein in 3 T3-L1, ALDH2-WT was expressed using the same plasmid backbone. (C) Sequencing analysis of the *ALDH2* gene exogenously expressed in 3 T3-L1 cells infected with ALDH2-WT (top) or ALDH2-G504 L-mut (bottom). (D) Expression of the transfected ALDH2 protein in 3 T3-L1 cells was indirectly assessed by the detection of RFP expression from the lentiviral vector. An RFP signal was detected by fluorescence microscopy at 48 h after infection in both 3 T3-L1 cells infected with ALDH2-WT and ALDH2-G504 L-mut. RFP control means 3 T3-L1 cells infected with plasmid backbone.

**Additional file 5: Table S1.** Information on the 27 clinical quantitative traits from 1999 populations.

**Additional file 6: Table S2.** Genetic correlation estimates, standard errors and *P* values for selected pairs of traits.

**Additional file 7: Table S3.** The information on essential genes correlated with more than 3 traits.

**Additional file 8: Table S4.** Twenty-nine SNPs (*P* < 1 × 10^− 4^) related to more than 3 traits were annotated in the HaploReg database.

**Additional file 9: Table S5.** The annotation of 31 (P < 1 × 10^− 4^) SNPs was associated with more than 1 trait.

**Additional file 10: Table S6.** The primer sequences of osteogenic and adipogenic differentiation in 3 T3-L1 cells.

## Data Availability

All data generated or analysed during this study are included in this published article [and its supplementary information files].

## Abbreviations

AFP: Alpha-fetoprotein
ALDH2: Aldehyde dehydrogenase 2 family member
ALT: Alanine transaminase
ASO: Anti-streptococcus haemolysin “O”
BUN: Blood urea nitrogen
C3: Complement 3
C4: Complement 4
CRP: C-Reactive protein
FAMHES: The Fangchenggang Area Male Health and Examination cohort
Fas: Fatty acids
FOL: Folate
FRRR: Ferritin
FSH: Follicle-stimulating hormone
GWAS: Genome-wide association studies
HCY: Homocysteine
HDL-C: High-density lipoprotein cholesterol
IgA: Immunoglobulin A
IgE: Immunoglobulin E
IgG: Immunoglobulin G
IgM: Immunoglobulin M
LDL-C: Low-density lipoprotein cholesterol
LDSC: Linkage disequilibrium score regression
MetS: Metabolic syndrome
OSTEOC: Osteocalcin
SNPs: Single nucleotide polymorphism
TE: Testosterone
TG: Triglyceride
UA: Uric acid
