# Supplementary figures and images for: A phenomics-based approach for the detection and interpretation of shared genetic influences on 29 biochemical indices in southern Chinese men
Source: BMC Genomics. 2019 Dec 16;20:983. doi: 10.1186/s12864-019-6363-0 (PMC6916074; doi:10.1186/s12864-019-6363-0)

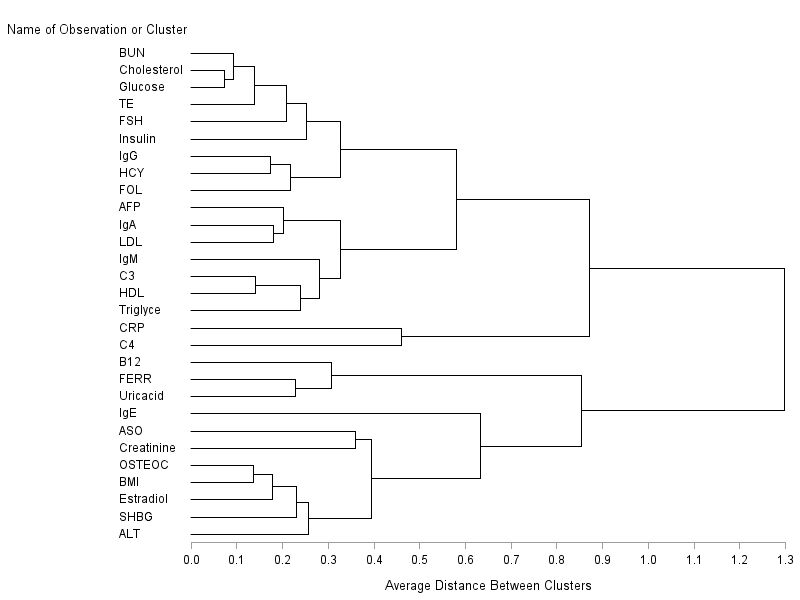

Supplement: Supplementary file 1 — Additional file 1: Fig. S1. The cluster dendrogram for the 29 biochemical indices from the FAMHES cohort created with the hclust win R package. In this analysis, two main clusters were produced among these 29 traits. FERR (ferritin), CRP (C-reactive protein), C3 (complement 3), C4 (complement 4), AFP (serum alpha-fetoprotein), TG (triglycerides), LDL (low density lipoprotein), ALT (alanine transaminase), BMI (body mass index), ASO (anti streptolysin) (anti-streptolysin “O”), IgG (immunoglobulin G), IgA (immunoglobulin A), IgM (immunoglobulin M), BUN (blood urea nitrogen), FSH (follicle-stimulating hormone), HDL (high-density lipoprotein), TE (testosterone), SHBG (sex hormone binding globulin), IgE (immunoglobulin E), B12 (vitamin B12), HCY (homocysteine). [file 12864_2019_6363_MOESM1_ESM.jpg]

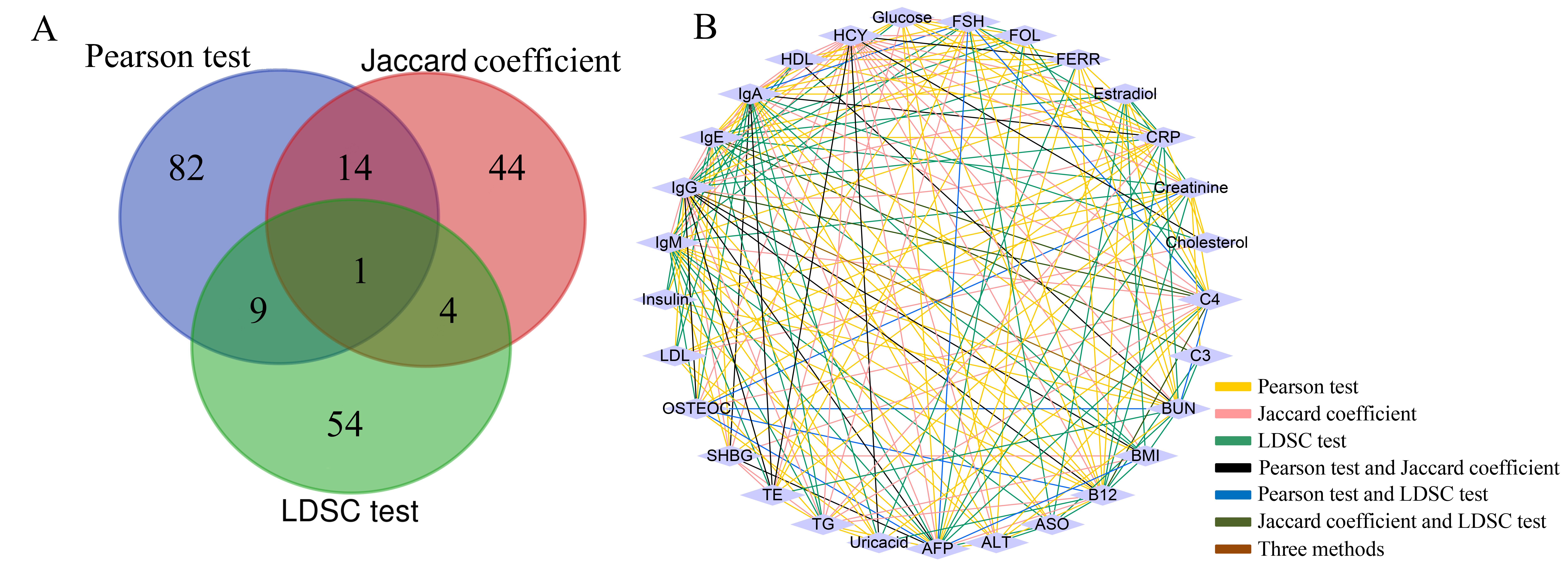

Supplement: Supplementary file 3 — Additional file 3: Fig. S3. The integration of correlated traits from three methods. (A) Venn diagram of the integration of correlated traits from three methods. (B) The related traits were integrated if they fulfilled the following conditions: the Pearson coefficient was greater than 0.3, the P value was less than 0.01, the Jaccard coefficient was greater than 0.6, or the LDSC p value was less than 0.05. Each testing method was denoted by a specific colour: green for Jaccard, and blue for LDSC. [file 12864_2019_6363_MOESM3_ESM.tif]

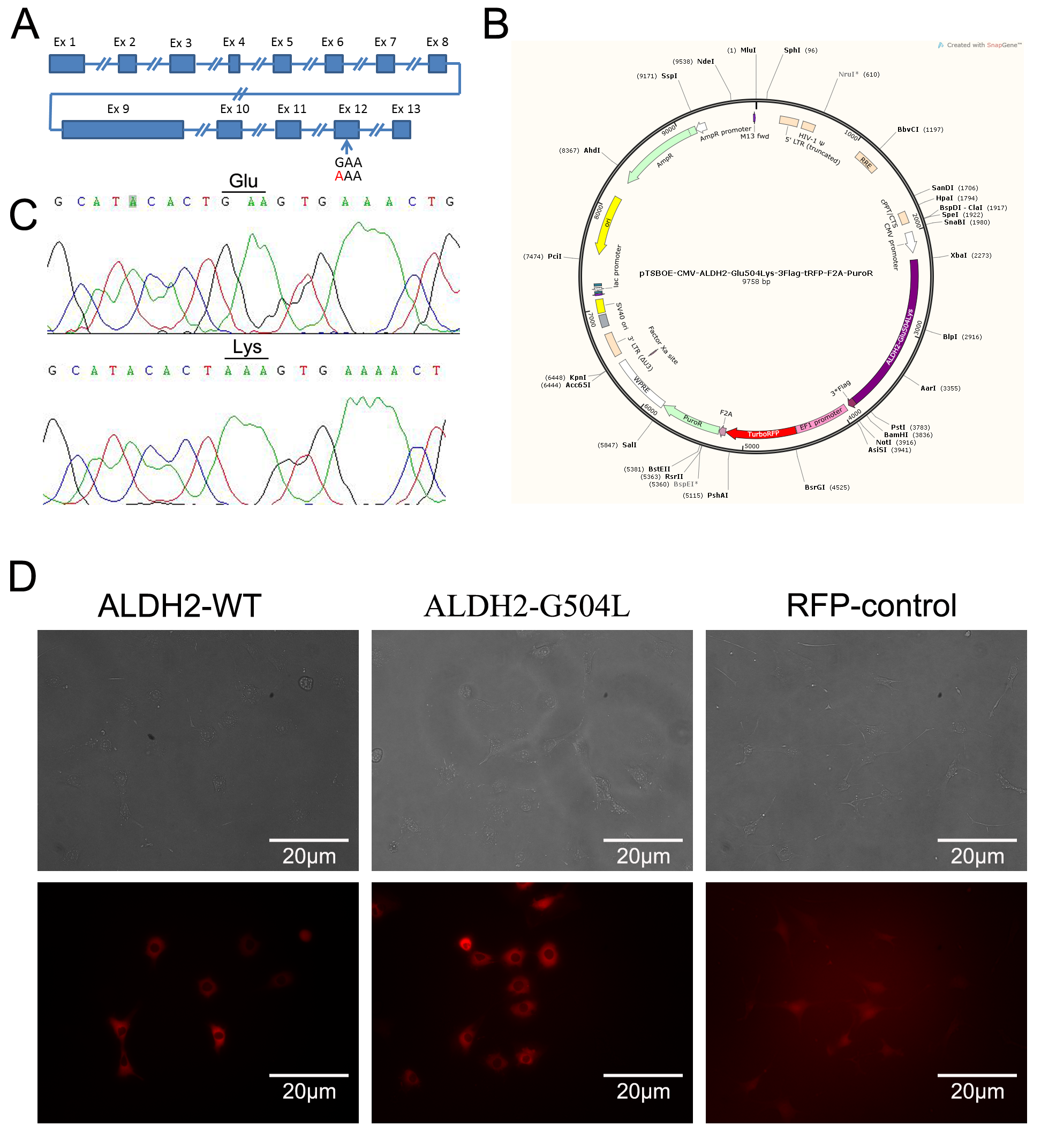

Supplement: Supplementary file 4 — Additional file 4: Fig. S4. A lentiviral vector was used to overexpress ALDH2-WT or ALDH2-G504 L-mut in 3 T3-L1 preadipocytes. (A) Localization of the Glu504Lys substitution mutation in ALDH2. Ex: exon. (B) The plasmid used to express the ALDH2-Gluc504Lys mutant protein in 3 T3-L1, ALDH2-WT was expressed using the same plasmid backbone. (C) Sequencing analysis of the ALDH2 gene exogenously expressed in 3 T3-L1 cells infected with ALDH2-WT (top) or ALDH2-G504 L-mut (bottom). (D) Expression of the transfected ALDH2 protein in 3 T3-L1 cells was indirectly assessed by the detection of RFP expression from the lentiviral vector. An RFP signal was detected by fluorescence microscopy at 48 h after infection in both 3 T3-L1 cells infected with ALDH2-WT and ALDH2-G504 L-mut. RFP control means 3 T3-L1 cells infected with plasmid backbone. [file 12864_2019_6363_MOESM4_ESM.tif]
