# Supplementary material for: A phenomics-based approach for the detection and interpretation of shared genetic influences on 29 biochemical indices in southern Chinese men
Source: BMC Genomics. 2019 Dec 16;20:983. doi: 10.1186/s12864-019-6363-0 (PMC6916074; doi:10.1186/s12864-019-6363-0)
Supplement: Supplementary file 5 — Additional file 5: Table S1. Information on the 27 clinical quantitative traits from 1999 populations. [file 12864_2019_6363_MOESM5_ESM.docx]

**Table S1.** The information of the 27 clinic quantitative traits from 1,999 populations.

| Clinical quantitative traits | Full name | Mean (95%CI) | Inflation factor |
| --- | --- | --- | --- |
| AFP(IU/ml) | alpha-fetoprotein | 2.56(2.26-2.85) | 1.015 |
| ALT(U/L) | glutamic-pyruvic transaminase | 47.19(42.54-51.84) | 1.017 |
| ASO(IU/ml) | antistreptolysin O | 79.44(65.55-93.34) | 1.017 |
| BMI | body mass index | 23.67(22.89-24.46) | 1.042 |
| BUN(mmol/L) | blood urea nitrogen | 5.34(5.06-5.61) | 1.047 |
| C3(g/L) | complement C3 | 1.16(1.10-1.22) | 1.046 |
| C4(mg/dl) | complement C4 | 0.35(0.32-0.38) | 1.054 |
| HCY(umol/L) | Homocysteine | 15.03(14.69-15.37) | 0.975 |
| Cholesterol(mmol/L) | cholesterol | 5.73(5.51-5.94) | 1.038 |
| Creatinine(μmoI/L) | creatinine | 87.04(84.23-89.84) | 1.017 |
| CRP(mg/L) | C-reactive protein | 1.67(0.72-2.62) | 1.016 |
| Estradiol(pg/ml) | estradiol | 35.52(32.97-38.08) | 1.025 |
| FSH(U/L) | follicle stimulating hormone | 5.86(4.95-6.77) | 1.029 |
| Glucose(mmol/L) | Blood glucose | 5.45(5.14-5.76) | 1.027 |
| HDL(mmol/L) | high density lipoprotein | 1.33(1.27-1.39) | 1.030 |
| IgA(g/L) | immunoglobulin A | 2.60(2.40-2.80) | 1.030 |
| IgE(IU/ml) | immunoglobulin E | 277.29(271.71-282.87) | 1.038 |
| IgG(g/L) | immunoglobulin G | 13.22(12.70-13.74) | 1.040 |
| IgM(g/L) | immunoglobulin M | 1.31(1.17-1.45) | 1.030 |
| Insulin(uU/ml) | insulin | 8.00(6.81-9.19) | 1.044 |
| OSTEOC(ng/L) | osteocalcin | 24.19(21.87-26.50) | 1.017 |
| LDL(mmol/L) | low density lipoprotein | 3.00(2.83-3.18) | 1.033 |
| B12(pg/ml) | vitamin B12 | 693.32(682.54-704.10) | 1.059 |
| SHBG(nmo1/L) | sex hormone binding globulin | 41.41(35.94-46.87) | 1.060 |
| TE(ng/ml) | testosterone | 6.17(5.67-6.67) | 1.014 |
| TG(mmol/L) | Triglyceride | 1.60(1.33-1.87) | 1.033 |
| Uricacid(umol/L) | uricacid | 401.68(383.22-420.14) | 1.018 |
| FOL(ng/ml) | folate | 9.44(9.31-9.58) | 1.029 |
| FERR(ng/ml) | ferritin | 365.55(355.68-375.62) | 1.008 |
