# Supplementary material for: A phenomics-based approach for the detection and interpretation of shared genetic influences on 29 biochemical indices in southern Chinese men
Source: BMC Genomics. 2019 Dec 16;20:983. doi: 10.1186/s12864-019-6363-0 (PMC6916074; doi:10.1186/s12864-019-6363-0)
Supplement: Supplementary file 6 — Additional file 6: Table S2. Genetic correlation estimates, standard errors and P values for selected pairs of traits. [file 12864_2019_6363_MOESM6_ESM.docx]

**Table S2.** Genetic correlation estimates, standard errors and P values for selected pairs of traits.

| Trait1 | Trait2 | Rg | Se | Z score | P value |
| --- | --- | --- | --- | --- | --- |
| ASO | AFP | 0.771 | 0.196 | 3.941 | 8.11E-05 |
| ASO | ALT | 0.724 | 0.326 | 2.223 | 0.026 |
| B12 | ASO | -0.566 | 0.141 | -4.017 | 5.91E-05 |
| BMI | AFP | 0.978 | 0.143 | 6.861 | 6.83E-12 |
| BUN | ASO | 0.566 | 0.215 | 2.629 | 9×10^-3^ |
| BUN | BMI | -0.684 | 0.143 | -4.768 | 1.86E-06 |
| C3 | B12 | -0.762 | 0.113 | -6.757 | 1.41E-11 |
| C3 | BUN | -0.311 | 0.127 | -2.447 | 0.014 |
| C4 | AFP | -0.225 | 0.107 | -2.106 | 0.035 |
| C4 | B12 | -0.173 | 0.067 | -2.577 | 0.010 |
| C4 | BUN | 0.594 | 0.260 | 2.283 | 0.022 |
| Creatinine | AFP | 0.683 | 0.166 | 4.119 | 3.81E-05 |
| Creatinine | ASO | -0.386 | 0.210 | -1.838 | 0.066 |
| Creatinine | B12 | -0.328 | 0.108 | -3.034 | 2×10^-3^ |
| CRP | AFP | 0.866 | 0.196 | 4.426 | 9.61E-06 |
| CRP | B12 | 0.861 | 0.152 | 5.679 | 1.36E-08 |
| CRP | BUN | 0.569 | 0.179 | 3.174 | 2×10^-3^ |
| Estradiol | ASO | -0.587 | 0.208 | -2.816 | 5×10^-3^ |
| Estradiol | BMI | 0.951 | 0.188 | 5.051 | 4.4E-07 |
| Estradiol | Creatinine | 0.925 | 0.230 | 4.020 | 5.81E-05 |
| Estradiol | CRP | -0.944 | 0.212 | -4.457 | 8.31E-06 |
| Estradiol | IgE | 0.388 | 0.153 | 2.527 | 0.012 |
| FOL | AFP | 0.513 | 0.162 | 3.176 | 2×10^-3^ |
| FOL | BMI | 0.293 | 0.135 | 2.176 | 0.030 |
| FOL | C4 | -0.424 | 0.150 | -2.822 | 5×10^-3^ |
| FSH | AFP | 0.681 | 0.165 | 4.125 | 3.71E-05 |
| FSH | ASO | -0.899 | 0.278 | -3.234 | 1×10^-3^ |
| FSH | C4 | 0.597 | 0.205 | 2.911 | 4×10^-3^ |
| FSH | CRP | -0.536 | 0.175 | -3.063 | 2×10^-3^ |
| FSH | FOL | 0.838 | 0.219 | 3.820 | 1.00E-04 |
| Glucose | IgG | 0.647 | 0.159 | 4.074 | 4.62E-05 |
| IgA | BMI | -0.683 | 0.139 | -4.901 | 9.55E-07 |
| IgA | Cholesterol | -0.997 | 0.179 | -5.583 | 2.36E-08 |
| IgA | FSH | -0.422 | 0.175 | -2.418 | 0.016 |
| IgE | AFP | 0.695 | 0.151 | 4.602 | 4.19E-06 |
| IgE | BMI | -0.544 | 0.118 | -4.593 | 4.37E-06 |
| IgE | C3 | -0.770 | 0.163 | -4.732 | 2.22E-06 |
| IgE | C4 | 0.510 | 0.173 | 2.940 | 3×10^-3^ |
| IgE | Creatinine | -0.547 | 0.162 | -3.373 | 1×10^-3^ |
| IgE | FOL | -0.382 | 0.161 | -2.381 | 0.017 |
| IgG | BUN | 0.761 | 0.175 | 4.339 | 1.43E-05 |
| IgG | C4 | 0.472 | 0.115 | 4.107 | 4.01E-05 |
| IgG | CRP | -0.335 | 0.140 | -2.391 | 0.017 |
| IgG | FERR | -0.289 | 0.140 | -2.069 | 0.039 |
| IgG | FSH | -0.568 | 0.158 | -3.597 | 3.00E-04 |
| IgG | HDL | -0.387 | 0.138 | -2.809 | 5×10^-3^ |
| IgM | AFP | -0.734 | 0.150 | -4.901 | 9.55E-07 |
| IgM | ASO | 0.823 | 0.202 | 4.078 | 4.55E-05 |
| IgM | Creatinine | -0.646 | 0.171 | -3.780 | 2.00E-04 |
| IgM | FSH | 0.607 | 0.162 | 3.750 | 2.00E-04 |
| IgM | HDL | 0.405 | 0.151 | 2.683 | 7×10^-3^ |
| Insulin | IgE | -0.541 | 0.114 | -4.747 | 2.07E-06 |
| Insulin | IgG | -0.581 | 0.113 | -5.155 | 2.54E-07 |
| LDL | IgA | -0.876 | 0.200 | -4.389 | 1.14E-05 |
| LDL | IgE | -0.491 | 0.150 | -3.270 | 1×10^-3^ |
| OSTEOC | AFP | -0.994 | 0.189 | -5.266 | 1.4E-07 |
| OSTEOC | B12 | -0.576 | 0.140 | -4.128 | 3.66E-05 |
| OSTEOC | BUN | -0.360 | 0.169 | -2.131 | 0.033 |
| OSTEOC | Creatinine | 0.849 | 0.209 | 4.071 | 4.69E-05 |
| OSTEOC | IgA | 0.537 | 0.184 | 2.921 | 4×10^-3^ |
| OSTEOC | IgM | 0.599 | 0.172 | 3.488 | 1×10^-3^ |
| TE | BUN | 0.477 | 0.208 | 2.295 | 0.022 |
| TE | IgM | 0.688 | 0.221 | 3.117 | 2×10^-3^ |
| TG | IgA | -0.786 | 0.163 | -4.822 | 1.42E-06 |
| TG | IgE | -0.791 | 0.145 | -5.444 | 5.2E-08 |
| TG | IgM | -0.318 | 0.117 | -2.728 | 6×10^-3^ |
| Uricacid | B12 | -0.914 | 0.228 | -4.012 | 6.01E-05 |
| Uricacid | FERR | 0.548 | 0.262 | 2.090 | 0.037 |
| Uricacid | IgA | 0.875 | 0.304 | 2.881 | 4×10^-3^ |

Rg: Estimates represent the average cross-trait LD Score regression estimates across 1,000 simulations;

Se: represents the mean cross-trait LD Score regression standard error across 1,000 simulations.
