# Supplementary material for: A phenomics-based approach for the detection and interpretation of shared genetic influences on 29 biochemical indices in southern Chinese men
Source: BMC Genomics. 2019 Dec 16;20:983. doi: 10.1186/s12864-019-6363-0 (PMC6916074; doi:10.1186/s12864-019-6363-0)
Supplement: Supplementary file 7 — Additional file 7: Table S3. The information on essential genes correlated with more than 3 traits. [file 12864_2019_6363_MOESM7_ESM.docx]

**Table S3.** The information on essential genes correlated with more than 3 traits.

| Number | Gene | Related Count | Related traits | Connect  genes |
| --- | --- | --- | --- | --- |
| 1 | APP | 4 | B12, Estradiol, TG, CRP | 634 |
| 2 | CCHCR1 | 4 | BMI, HCY, Creatinine, C4 | 21 |
| 3 | PDE4D | 3 | FSH, SHBG, OSTEOC | 11 |
| 4 | HLA-C | 3 | C4, IgM, Creatinine | 30 |
| 5 | AIF1 | 3 | ASO, Creatinine, C4 | 1 |
| 6 | AJAP1 | 4 | B12, TG, HCY, Creatinine | 1 |
| 7 | ALDH2 | 9 | ALT, Glucose, TG, TE, SHBG, OSTEOC, FOL, FERR, BMI | 19 |
| 8 | FXR2 | 5 | IgA, TE, SHBG, IgM, IgG | 42 |
| 9 | NAV3 | 3 | HCY, IgE, HDL | 3 |
| 10 | LRP8 | 3 | HCY, LDL, IgE | 13 |
| 11 | MEOX2 | 4 | C4, IgG, HDL, HCY | 80 |
| 12 | CDH13 | 6 | AFP, TG, TE, IgM, BUN, BMI | 3 |
| 13 | AUTS2 | 3 | HDL, TE, SHBG | 10 |
| 14 | NCK2 | 3 | Creatinine, IgM, HDL | 48 |
| 15 | CNTN4 | 3 | HCY, IgE, HDL | 2 |
| 16 | NETO1 | 3 | CRP, Uricacid, HDL | 1 |
| 17 | ITPR1 | 3 | ALT, Insulin, Estradiol | 17 |
| 18 | CSMD1 | 8 | C4, Insulin, Uricacid, LDL, HCY, FERR, Creatinine, Cholesterol | 1 |
| 19 | ACTL8 | 3 | BMI, Insulin, SHBG | 8 |
| 20 | BRAP | 9 | ALT, Glucose, TG, TE, SHBG, OSTEOC, FOL, FERR, BMI | 13 |
| 21 | BRD2 | 3 | BUN, CRP, C4 | 8 |
| 22 | NEDD1 | 3 | ASO, IgG, IgA | 42 |
| 23 | TBX3 | 4 | BUN, LDL, IgM, Cholesterol | 9 |
| 24 | CACNA2D1 | 3 | AFP, IgM, FERR | 10 |
| 25 | RAPGEF2 | 4 | C3, Estradiol, HCY, Cholesterol | 27 |
| 26 | SLC39A11 | 3 | C4, Estradiol, HCY | 4 |
| 27 | ITGB3 | 3 | HDL, TE, SHBG | 6 |
| 28 | DAB1 | 4 | B12, FSH, CRP, C4 | 20 |
| 29 | SENP3 | 5 | IgA, TE, SHBG, IgM, IgG | 35 |
| 30 | SOX5 | 3 | BMI, IgM, HCY | 15 |
| 31 | SKIV2L | 3 | ASO, C4, C3 | 9 |
| 32 | POLD3 | 3 | ALT, IgE, CRP | 7 |
| 33 | GPC6 | 4 | IgE, Uricacid, IgM, IgG | 4 |
| 34 | DUSP10 | 3 | C4, Insulin, FERR | 8 |
| 35 | HLA-DRB1 | 3 | C3, Creatinine, C4 | 7 |
| 36 | CNTNAP2 | 4 | AFP, Insulin, BUN, ASO | 4 |
| 37 | CNTNAP4 | 3 | BMI, Glucose, Uricacid | 5 |
| 38 | PARK2 | 4 | FSH, IgA, HDL, HCY | 129 |
| 39 | LMO4 | 3 | C4, HCY, FSH | 21 |
| 40 | KBTBD8 | 3 | B12, IgM, HCY | 1 |
| 41 | UNC5C | 3 | IgG, Glucose, Uricacid | 3 |
| 42 | SRGAP3 | 3 | Cholesterol, IgE, HCY | 8 |
| 43 | CTNNA2 | 3 | Creatinine, HCY, FERR | 4 |
| 44 | DOCK4 | 4 | B12, Uricacid, TG, HCY | 5 |
| 45 | NDN | 4 | C4, Uricacid, TG, IgM | 21 |
| 46 | POU5F1 | 3 | BMI, HCY, C4 | 95 |
| 47 | CDKAL1 | 3 | AFP, IgG, ASO | 4 |
| 48 | LRP1B | 5 | BMI, IgM, IgG, IgA, FSH | 4 |
| 49 | NTRK3 | 4 | CRP, Estradiol, HCY | 6 |
| 50 | FTO | 3 | B12, HCY, BMI | 5 |
| 51 | IRF8 | 3 | B12, C4, BMI | 5 |
| 52 | MYL2 | 4 | ALT, TG, TE, Cholesterol | 1 |
| 53 | RELN | 3 | C3, Insulin, HCY, | 2 |
| 54 | ZWINT | 4 | FSH, Estradiol, SHBG, HCY | 29 |
| 55 | PRKG1 | 3 | BMI, Estradiol, C3 | 9 |
| 56 | CTNND2 | 3 | IgE, Estradiol, OSTEOC | 4 |
| 57 | TH | 4 | C4, LDL, FOL, Cholesterol | 3 |
| 58 | PSD3 | 3 | ASO, IgA, Creatinine | 2 |
| 59 | SNX19 | 3 | HCY, Glucose, TG | 1 |
| 60 | NPAS3 | 4 | ASO, TE, SHBG, BMI | 2 |
| 61 | LRRC4C | 3 | B12, Uricacid, HCY | 1 |
| 62 | RPH3A | 3 | C3, TG, OSTEOC | 2 |
| 63 | NUDT12 | 3 | AFP, HCY, C4 | 4 |
| 64 | RGS7 | 3 | ASO, Creatinine, Cholesterol | 2 |
| 65 | PTPRU | 3 | ALT, HCY, Creatinine | 1 |
| 66 | THSD7A | 3 | FSH, Glucose, HCY | 1 |
| 67 | EBF2 | 3 | Cholesterol, IgG, HCY | 2 |
| 68 | RGS6 | 3 | FOL, OSTEOC, HCY | 1 |
| 69 | DMRT2 | 4 | BMI, SHBG, Creatinine, CRP | 1 |
| 70 | IRX1 | 3 | BMI, SHBG, HCY | 1 |
| 71 | NKX2-3 | 3 | C4, Glucose, FOL | 1 |
