# Supplementary material for: A phenomics-based approach for the detection and interpretation of shared genetic influences on 29 biochemical indices in southern Chinese men
Source: BMC Genomics. 2019 Dec 16;20:983. doi: 10.1186/s12864-019-6363-0 (PMC6916074; doi:10.1186/s12864-019-6363-0)
Supplement: Supplementary file 8 — Additional file 8: Table S4. Twenty-nine SNPs (P < 1 × 10− 4) related to more than 3 traits were annotated in the HaploReg database. [file 12864_2019_6363_MOESM8_ESM.docx]

**Table S4.** 29 SNPs (P<1✕10^-4^) related to more than 3 traits were annotated in the HaploReg database.

| SNP | Genes | Traits | DNase | Enhancer histone marks | eQTL(p-value) | Region |
| --- | --- | --- | --- | --- | --- | --- |
| rs10774609 | CCDC63 | ALT, Cholesterol, TG |  | H3K4me1, H3K27ac | PPP1CC(1×10^-3^) | INT |
| rs11065756 | CCDC63 | ALT, Cholesterol, TG | IPSC, MUS, BRN | H3K4me1 |  | INT |
| rs11065766 | MYL2 | ALT, Cholesterol, TG | BLD | H3K4me1, H3K27ac | PPP1CC(2×10^-3^) | INT |
| rs11065770 | MYL2 | ALT, Cholesterol, TG | BLD | H3K4me1, H3K27ac |  | INT |
| rs11065773 | MYL2 | ALT, Cholesterol, TG | SKIN | H3K4me1, |  | INT |
| rs11065774 | MYL2 | ALT, Cholesterol, TG | IPSC, SKIN, HRT, GI, MUS, BRN | H3K4me1, H3K27ac |  | INT |
| rs11116969 | AC021887.1 | SHBG, TE, TG |  |  | NTS(2.313E-06), RASSF9(6.377E-13) |  |
| rs1115245 | C9orf92 | Uricacid, SHBG, TE, TG |  | H3K27ac |  |  |
| rs11610779 | CCDC63 | ALT, Cholesterol, TG |  | H3K4me1, H3K27ac | PPP1CC(0.002) | INT |
| rs11929794 | SNORD65 | BMI, C3, SHBG |  |  |  |  |
| rs12229654 | RP1-46F2.2 | ALT, BMI, FERR, Glucose, OSTEOC, SHBG, TE, TG |  |  |  |  |
| rs12231049 | MYL2 | ALT, Cholesterol, TG | HRT | H3K4me1, H3K27ac |  | INT |
| rs2071629 | MYL2 | ALT, Cholesterol, TG | BLD | H3K4me1, H3K27ac |  | INT |
| rs2188380 | RP1-46F2.2 | ALT, BMI, FERR, Glucose, OSTEOC, SHBG, TE, TG | ESDR, SKIN, PLCNT, BRST | H3K4me1, H3K27ac |  |  |
| rs3782886 | BRAP | ALT, BMI, FERR, FOL, Glucose, OSTEOC, SHBG, TE, TG | BLD | H3K4me1, H3K27ac |  | SYN |
| rs3782888 | MYL2 | ALT, Cholesterol, TG | ESDR, IPSC, BRST, SKIN, HRT, GI, LNG, CRVX, MUS, VAS, BRN | H3K4me1, H3K27ac |  | INT |
| rs3803800 | TNFSF13 | IgA, IgG, IgM | ESC, ESDR, BLD, GI, OVRY, PANC | H3K4me1, H3K27ac | EIF4A1\|CD68(4.719E-9), SAT2(3.415E-4), TNFSF12(2.055E-4), TNFSF12(7.342E-14) | NSM;INT |
| rs3809297 | CUX2 | ALT, BMI, FERR, Glucose, OSTEOC, SHBG, TE, TG | BRN | H3K4me1, H3K27ac |  | INT |
| rs3825389 | MYL2 | ALT, Cholesterol, TG |  | H3K4me1, H3K27ac |  | INT |
| rs4602096 | SENP3 | IgA, IgG, IgM | MUS, OVRY, BLD, SKIN | H3K4me1, H3K27ac | EIF4A1\|CD68(2.788E-22), TNFSF12(1.028E-14), TNFSF12\|EIF4A1(1×10^-3^) | INT |
| rs4646776 | ALDH2 | ALT, BMI, FERR, FOL, Glucose, OSTEOC, SHBG, TE, TG | ADRL, OVRY | H3K4me1, H3K27ac |  | INT |
| rs4766566 | CUX2 | ALT, Glucose, OSTEOC, TG | IPSC, BLD | H3K4me1, H3K27ac | FAM109A(5.239E-06), ATXN2(9.804E-5), FAM109A(8.666E-4), SH2B3(1.982E-8) | INT |
| rs6027570 | RP5-1043L13.1 | Cholesterol, HCY, LDL |  |  |  |  |
| rs6489822 | CCDC63 | ALT, Cholesterol, TG | BRST, SKIN | H3K4me1, H3K27ac | PPP1CC(2×10^-3^) | INT |
| rs6490029 | CUX2 | ALT, Glucose, OSTEOC |  | H3K4me1, H3K27ac | SH2B3(1.314E-6) | INT |
| rs6535256 | RP11-689K5.3 | BMI, Insulin, TG |  | H3K4me1, H3K27ac |  |  |
| rs671 | ALDH2 | ALT, BMI, FERR, FOL, Glucose, OSTEOC, SHBG, TE, TG |  | H3K4me1, H3K27ac |  | NSM |
| rs7488411 | MYL2 | ALT, Cholesterol, TG |  | H3K4me1, H3K27ac |  | INT |
| rs916682 | CUX2 | ALT, Glucose, OSTEOC, TG |  | H3K4me1, H3K27ac | FAM109A(7.176E-06) | INT |

Notes: INT: intron. SYN: synonymous. NSM: missense
