# Supplementary material for: A phenomics-based approach for the detection and interpretation of shared genetic influences on 29 biochemical indices in southern Chinese men
Source: BMC Genomics. 2019 Dec 16;20:983. doi: 10.1186/s12864-019-6363-0 (PMC6916074; doi:10.1186/s12864-019-6363-0)
Supplement: Supplementary file 9 — Additional file 9: Table S5. The annotation of 31 (P < 1 × 10− 4) SNPs was associated with more than 1 trait. [file 12864_2019_6363_MOESM9_ESM.docx]

**Table S5.** The annotation of 31(P<1✕10^-4^) SNPs associated with more than 1 trait.

| CHR | Position | SNP | Gene | Type |
| --- | --- | --- | --- | --- |
| 1 | 11778965 | rs1801133 | MTHFR | FOL, HCY, Homocysteine levels (1) |
| 5 | 74672240 | rs10045497 | HMGCR | Cholesterol, LDL, Lipid traits (2) |
| 6 | 31886508 | rs2075799 | HSPA1L | ASO, C4, Complement C3 and C4 levels (3) |
| 6 | 32471933 | rs2076529 | BTNL2 | C3, C4, Waist-hip ratio (4) |
| 6 | 32038330 | rs2734331 | SKIV2L | ASO, C4, Blood metabolite levels (5) |
| 9 | 135143989 | rs579459 | ABO | Cholesterol, LDL, Blood metabolite ratios (5), Urinary metabolites (H-NMR features) (6), Coronary artery disease or ischemic stroke (7), Coronary artery disease (7), Coronary artery disease or large artery stroke (7), Red blood cell traits (8), Liver enzyme levels (alkaline phosphatase) (9), Coronary heart disease (9), Soluble levels of adhesion molecules (10), Soluble E-selectin levels (11) |
| 9 | 135144125 | rs649129 | ABO | Cholesterol, LDL, Blood metabolite ratios (5), Soluble levels of adhesion molecules (12) |
| 9 | 135139220 | rs507666 | ABO | Cholesterol, LDL, Lipid traits (2) |
| 9 | 135144688 | rs495828 | ABO | Cholesterol, LDL, Blood metabolite ratios (5) |
| 9 | 135143696 | rs651007 | ABO | Cholesterol, LDL, Iron status biomarkers (ferritin levels) (13), Blood metabolite levels (5), Serum alkaline phosphatase levels (14), End-stage coagulation (15), Metabolite levels (16), E-selectin levels (17) |
| 12 | 109898844 | rs12229654 | CUX2 | ALT, FERR, Glucose, OSTEOC, SHBG, TE, TG Metabolic syndrome (18), BMI (19), Glycemic traits (20), HDL Cholesterol (16), Gamma glutamyl transpeptidase (16), Alcohol consumption (21) |
| 12 | 109870510 | rs2188380 | CUX2 | ALT, BMI, FERR, Glucose, OSTEOC, SHBG, TG, TE, Gout (22) |
| 12 | 110594872 | rs3782886 | BRAP | ALT, BMI, FERR, FOL, Glucose, OSTEOC, SHBG, TE, TG, Myocardial infarction (23), Hematological and biochemical traits (24) |
| 12 | 110726149 | rs671 | ALDH2 | ALT, BMI, FERR, FOL, Glucose, OSTEOC, SHBG, TE, TG, Body mass index (19), Alcohol consumption (maxi-drinks) (25), Response to alcohol consumption (flushing response) (25), Alcohol dependence (25), Renal function-related traits (sCR) (26), Intracranial aneurysm (27), Triglycerides (28), Coronary heart disease (29), Drinking behavior (30), Hematological and biochemical (24), Mean corpuscular hemoglobin concentration (24), Esophageal cancer (31) |
| 12 | 109824626 | rs10774610 | CCDC63 | ALT, Glucose, TG, Drinking behavior (30) |
| 12 | 109818005 | rs10849915 | CCDC63 | ALT, TG, Alcohol consumption (21) |
| 12 | 111302166 | rs11066280 | RPL6 | FERR, OSTEOC, Cholesterol, Metabolic syndrome (18), Systolic blood pressure (32), Diastolic blood pressure (32), Hypertension (32), Drinking behavior (30), Coronary heart disease (32), Metabolite levels (16), Esophageal cancer (33), Blood pressure (34) |
| 12 | 111129784 | rs2074356 | C12orf51 | OSTEOC, TG, Glycemic traits (20), Renal function-related traits (BUN) (26), Gamma glutamyl transpeptidase (16), HDL Cholesterol (16), Esophageal cancer (33), Alcohol consumption (21), Biomedical quantitative traits (35) |
| 12 | 109835038 | rs3782889 | MYL2 | TG, ALT, Coronary heart disease (36) |
| 15 | 49386975 | rs10519302 | CYP19A1 | Estradiol, FSH, Height (37) |
| 15 | 56470658 | rs1532085 | LIPC | Cholesterol, HDL Cholesterol (16), TG (28), Cholesterol, total (38), Red blood cell traits (8), Metabolite levels (16), Metabolic syndrome (39), HDL Cholesterol (40), Cholesterol, total (40), Lipid metabolism phenotypes (41), HDL Cholesterol (42), Cholesterol (42), HDL Cholesterol (43) |
| 15 | 49317787 | rs16964211 | CYP19A1 | Cholesterol, Height (44, 45) |
| 15 | 49356702 | rs2305707 | CYP19A1 | Estradiol, FSH, Height (26) |
| 15 | 49311584 | rs2414095 | CYP19A1 | Estradiol, FSH (46), Hormone measurements (46) |
| 15 | 49301021 | rs6493487 | CYP19A1 | Estradiol, FSH, Thiazide-induced adverse metabolic effects in hypertensive patients (47) |
| 15 | 49321839 | rs727479 | CYP19A1 | Estradiol, FSH, Estradiol levels (48), |
| 17 | 7403693 | rs3803800 | TNFSF13 | IgM, IgG, IgA nephropathy (49), IgA levels (50), Non-albumin protein levels (51), IgA nephropathy (52) |
| 17 | 7409953 | rs11078697 | SENP3 | IgM, SHBG, IgM levels (53) |
| 17 | 7452799 | rs11653545 | FXR2 | IgM, SHBG, IgM levels (53) |
| 19 | 50107480 | rs445925 | LOC100129500 | Cholesterol, LDL, Response to statins (LDL Cholesterol change) (54), Blood metabolite ratios (5), Lipoprotein-associated phospholipase A2 activity and mass (55), Apolipoprotein Levels (56), Metabolite levels(57), Carotid intima media thickness (58), Cardiovascular disease risk factors (59) |

**Reference:**

1. van Meurs JB, Pare G, Schwartz SM, Hazra A, Tanaka T, Vermeulen SH, Cotlarciuc I, Yuan X, Malarstig A, Bandinelli S*, et al.* (2013) Common genetic loci influencing plasma homocysteine concentrations and their effect on risk of coronary artery disease. *Am J Clin Nutr* 98(3):668-676.

2. Zhou L, He M, Mo Z, Wu C, Yang H, Yu D, Yang X, Zhang X, Wang Y, Sun J*, et al.* (2013) A genome wide association study identifies common variants associated with lipid levels in the Chinese population. *PLoS One* 8(12):e82420.

3. Yang X, Sun J, Gao Y, Tan A, Zhang H, Hu Y, Feng J, Qin X, Tao S, Chen Z*, et al.* (2012) Genome-wide association study for serum complement C3 and C4 levels in healthy Chinese subjects. *PLoS Genet* 8(9):e1002916.

4. Heid IM, Jackson AU, Randall JC, Winkler TW, Qi L, Steinthorsdottir V, Thorleifsson G, Zillikens MC, Speliotes EK, Magi R*, et al.* (2010) Meta-analysis identifies 13 new loci associated with waist-hip ratio and reveals sexual dimorphism in the genetic basis of fat distribution. *Nat Genet* 42(11):949-960.

5. Shin SY, Fauman EB, Petersen AK, Krumsiek J, Santos R, Huang J, Arnold M, Erte I, Forgetta V, Yang TP*, et al.* (2014) An atlas of genetic influences on human blood metabolites. *Nat Genet* 46(6):543-550.

6. Rueedi R, Ledda M, Nicholls AW, Salek RM, Marques-Vidal P, Morya E, Sameshima K, Montoliu I, Da Silva L, Collino S*, et al.* (2014) Genome-wide association study of metabolic traits reveals novel gene-metabolite-disease links. *PLoS Genet* 10(2):e1004132.

7. Dichgans M, Malik R, Konig IR, Rosand J, Clarke R, Gretarsdottir S, Thorleifsson G, Mitchell BD, Assimes TL, Levi C*, et al.* (2014) Shared genetic susceptibility to ischemic stroke and coronary artery disease: a genome-wide analysis of common variants. *Stroke* 45(1):24-36.

8. van der Harst P, Zhang W, Mateo Leach I, Rendon A, Verweij N, Sehmi J, Paul DS, Elling U, Allayee H, Li X*, et al.* (2012) Seventy-five genetic loci influencing the human red blood cell. *Nature* 492(7429):369-375.

9. Schunkert H, Konig IR, Kathiresan S, Reilly MP, Assimes TL, Holm H, Preuss M, Stewart AF, Barbalic M, Gieger C*, et al.* (2011) Large-scale association analysis identifies 13 new susceptibility loci for coronary artery disease. *Nat Genet* 43(4):333-338.

10. Chambers JC, Zhang W, Sehmi J, Li X, Wass MN, Van der Harst P, Holm H, Sanna S, Kavousi M, Baumeister SE*, et al.* (2011) Genome-wide association study identifies loci influencing concentrations of liver enzymes in plasma. *Nat Genet* 43(11):1131-1138.

11. Paterson AD, Lopes-Virella MF, Waggott D, Boright AP, Hosseini SM, Carter RE, Shen E, Mirea L, Bharaj B, Sun L*, et al.* (2009) Genome-wide association identifies the ABO blood group as a major locus associated with serum levels of soluble E-selectin. *Arterioscler Thromb Vasc Biol* 29(11):1958-1967.

12. Barbalic M, Dupuis J, Dehghan A, Bis JC, Hoogeveen RC, Schnabel RB, Nambi V, Bretler M, Smith NL, Peters A*, et al.* (2010) Large-scale genomic studies reveal central role of ABO in sP-selectin and sICAM-1 levels. *Hum Mol Genet* 19(9):1863-1872.

13. Benyamin B, Esko T, Ried JS, Radhakrishnan A, Vermeulen SH, Traglia M, Gogele M, Anderson D, Broer L, Podmore C*, et al.* (2014) Novel loci affecting iron homeostasis and their effects in individuals at risk for hemochromatosis. *Nat Commun* 5:4926.

14. Li J, Gui L, Wu C, He Y, Zhou L, Guo H, Yuan J, Yang B, Dai X, Deng Q*, et al.* (2013) Genome-wide association study on serum alkaline phosphatase levels in a Chinese population. *BMC Genomics* 14:684.

15. Williams FM, Carter AM, Hysi PG, Surdulescu G, Hodgkiss D, Soranzo N, Traylor M, Bevan S, Dichgans M, Rothwell PM*, et al.* (2013) Ischemic stroke is associated with the ABO locus: the EuroCLOT study. *Ann Neurol* 73(1):16-31.

16. Kim YJ, Go MJ, Hu C, Hong CB, Kim YK, Lee JY, Hwang JY, Oh JH, Kim DJ, Kim NH*, et al.* (2011) Large-scale genome-wide association studies in East Asians identify new genetic loci influencing metabolic traits. *Nat Genet* 43(10):990-995.

17. Qi L, Cornelis MC, Kraft P, Jensen M, van Dam RM, Sun Q, Girman CJ, Laurie CC, Mirel DB, Hunter DJ*, et al.* (2010) Genetic variants in ABO blood group region, plasma soluble E-selectin levels and risk of type 2 diabetes. *Hum Mol Genet* 19(9):1856-1862.

18. Shim U, Kim HN, Sung YA, & Kim HL (2014) Pathway Analysis of Metabolic Syndrome Using a Genome-Wide Association Study of Korea Associated Resource (KARE) Cohorts. *Genomics Inform* 12(4):195-202.

19. Wen W, Zheng W, Okada Y, Takeuchi F, Tabara Y, Hwang JY, Dorajoo R, Li H, Tsai FJ, Yang X*, et al.* (2014) Meta-analysis of genome-wide association studies in East Asian-ancestry populations identifies four new loci for body mass index. *Hum Mol Genet* 23(20):5492-5504.

20. Go MJ, Hwang JY, Kim YJ, Hee Oh J, Kim YJ, Heon Kwak S, Soo Park K, Lee J, Kim BJ, Han BG*, et al.* (2013) New susceptibility loci in MYL2, C12orf51 and OAS1 associated with 1-h plasma glucose as predisposing risk factors for type 2 diabetes in the Korean population. *J Hum Genet* 58(6):362-365.

21. Baik I, Cho NH, Kim SH, Han BG, & Shin C (2011) Genome-wide association studies identify genetic loci related to alcohol consumption in Korean men. *Am J Clin Nutr* 93(4):809-816.

22. Matsuo H, Yamamoto K, Nakaoka H, Nakayama A, Sakiyama M, Chiba T, Takahashi A, Nakamura T, Nakashima H, Takada Y*, et al.* (2016) Genome-wide association study of clinically defined gout identifies multiple risk loci and its association with clinical subtypes. *Ann Rheum Dis* 75(4):652-659.

23. Hirokawa M, Morita H, Tajima T, Takahashi A, Ashikawa K, Miya F, Shigemizu D, Ozaki K, Sakata Y, Nakatani D*, et al.* (2015) A genome-wide association study identifies PLCL2 and AP3D1-DOT1L-SF3A2 as new susceptibility loci for myocardial infarction in Japanese. *Eur J Hum Genet* 23(3):374-380.

24. Kamatani Y, Matsuda K, Okada Y, Kubo M, Hosono N, Daigo Y, Nakamura Y, & Kamatani N (2010) Genome-wide association study of hematological and biochemical traits in a Japanese population. *Nat Genet* 42(3):210-215.

25. Quillen EE, Chen XD, Almasy L, Yang F, He H, Li X, Wang XY, Liu TQ, Hao W, Deng HW*, et al.* (2014) ALDH2 is associated to alcohol dependence and is the major genetic determinant of "daily maximum drinks" in a GWAS study of an isolated rural Chinese sample. *Am J Med Genet B Neuropsychiatr Genet* 165b(2):103-110.

26. Okada Y, Sim X, Go MJ, Wu JY, Gu D, Takeuchi F, Takahashi A, Maeda S, Tsunoda T, Chen P*, et al.* (2012) Meta-analysis identifies multiple loci associated with kidney function-related traits in east Asian populations. *Nat Genet* 44(8):904-909.

27. Low SK, Takahashi A, Cha PC, Zembutsu H, Kamatani N, Kubo M, & Nakamura Y (2012) Genome-wide association study for intracranial aneurysm in the Japanese population identifies three candidate susceptible loci and a functional genetic variant at EDNRA. *Hum Mol Genet* 21(9):2102-2110.

28. Tan A, Sun J, Xia N, Qin X, Hu Y, Zhang S, Tao S, Gao Y, Yang X, Zhang H*, et al.* (2012) A genome-wide association and gene-environment interaction study for serum triglycerides levels in a healthy Chinese male population. *Hum Mol Genet* 21(7):1658-1664.

29. Takeuchi F, Yokota M, Yamamoto K, Nakashima E, Katsuya T, Asano H, Isono M, Nabika T, Sugiyama T, Fujioka A*, et al.* (2012) Genome-wide association study of coronary artery disease in the Japanese. *Eur J Hum Genet* 20(3):333-340.

30. Takeuchi F, Isono M, Nabika T, Katsuya T, Sugiyama T, Yamaguchi S, Kobayashi S, Ogihara T, Yamori Y, Fujioka A*, et al.* (2011) Confirmation of ALDH2 as a Major locus of drinking behavior and of its variants regulating multiple metabolic phenotypes in a Japanese population. *Circ J* 75(4):911-918.

31. Cui R, Kamatani Y, Takahashi A, Usami M, Hosono N, Kawaguchi T, Tsunoda T, Kamatani N, Kubo M, Nakamura Y*, et al.* (2009) Functional variants in ADH1B and ALDH2 coupled with alcohol and smoking synergistically enhance esophageal cancer risk. *Gastroenterology* 137(5):1768-1775.

32. Lu X, Wang L, Lin X, Huang J, Charles Gu C, He M, Shen H, He J, Zhu J, Li H*, et al.* (2015) Genome-wide association study in Chinese identifies novel loci for blood pressure and hypertension. *Hum Mol Genet* 24(3):865-874.

33. Wu C, Hu Z, He Z, Jia W, Wang F, Zhou Y, Liu Z, Zhan Q, Liu Y, Yu D*, et al.* (2011) Genome-wide association study identifies three new susceptibility loci for esophageal squamous-cell carcinoma in Chinese populations. *Nat Genet* 43(7):679-684.

34. Kato N, Takeuchi F, Tabara Y, Kelly TN, Go MJ, Sim X, Tay WT, Chen CH, Zhang Y, Yamamoto K*, et al.* (2011) Meta-analysis of genome-wide association studies identifies common variants associated with blood pressure variation in east Asians. *Nat Genet* 43(6):531-538.

35. Cho YS, Go MJ, Kim YJ, Heo JY, Oh JH, Ban HJ, Yoon D, Lee MH, Kim DJ, Park M*, et al.* (2009) A large-scale genome-wide association study of Asian populations uncovers genetic factors influencing eight quantitative traits. *Nat Genet* 41(5):527-534.

36. Lee JY, Lee BS, Shin DJ, Woo Park K, Shin YA, Joong Kim K, Heo L, Young Lee J, Kyoung Kim Y, Jin Kim Y*, et al.* (2013) A genome-wide association study of a coronary artery disease risk variant. *J Hum Genet* 58(3):120-126.

37. He M, Xu M, Zhang B, Liang J, Chen P, Lee JY, Johnson TA, Li H, Yang X, Dai J*, et al.* (2015) Meta-analysis of genome-wide association studies of adult height in East Asians identifies 17 novel loci. *Hum Mol Genet* 24(6):1791-1800.

38. Willer CJ, Schmidt EM, Sengupta S, Peloso GM, Gustafsson S, Kanoni S, Ganna A, Chen J, Buchkovich ML, Mora S*, et al.* (2013) Discovery and refinement of loci associated with lipid levels. *Nat Genet* 45(11):1274-1283.

39. Kristiansson K, Perola M, Tikkanen E, Kettunen J, Surakka I, Havulinna AS, Stancakova A, Barnes C, Widen E, Kajantie E*, et al.* (2012) Genome-wide screen for metabolic syndrome susceptibility Loci reveals strong lipid gene contribution but no evidence for common genetic basis for clustering of metabolic syndrome traits. *Circ Cardiovasc Genet* 5(2):242-249.

40. Teslovich TM, Musunuru K, Smith AV, Edmondson AC, Stylianou IM, Koseki M, Pirruccello JP, Ripatti S, Chasman DI, Willer CJ*, et al.* (2010) Biological, clinical and population relevance of 95 loci for blood lipids. *Nature* 466(7307):707-713.

41. Chasman DI, Pare G, Mora S, Hopewell JC, Peloso G, Clarke R, Cupples LA, Hamsten A, Kathiresan S, Malarstig A*, et al.* (2009) Forty-three loci associated with plasma lipoprotein size, concentration, and cholesterol content in genome-wide analysis. *PLoS Genet* 5(11):e1000730.

42. Aulchenko YS, Ripatti S, Lindqvist I, Boomsma D, Heid IM, Pramstaller PP, Penninx BW, Janssens AC, Wilson JF, Spector T*, et al.* (2009) Loci influencing lipid levels and coronary heart disease risk in 16 European population cohorts. *Nat Genet* 41(1):47-55.

43. Sabatti C, Service SK, Hartikainen AL, Pouta A, Ripatti S, Brodsky J, Jones CG, Zaitlen NA, Varilo T, Kaakinen M*, et al.* (2009) Genome-wide association analysis of metabolic traits in a birth cohort from a founder population. *Nat Genet* 41(1):35-46.

44. Wood AR, Esko T, Yang J, Vedantam S, Pers TH, Gustafsson S, Chu AY, Estrada K, Luan J, Kutalik Z*, et al.* (2014) Defining the role of common variation in the genomic and biological architecture of adult human height. *Nat Genet* 46(11):1173-1186.

45. Lango Allen H, Estrada K, Lettre G, Berndt SI, Weedon MN, Rivadeneira F, Willer CJ, Jackson AU, Vedantam S, Raychaudhuri S*, et al.* (2010) Hundreds of variants clustered in genomic loci and biological pathways affect human height. *Nature* 467(7317):832-838.

46. Chen Z, Tao S, Gao Y, Zhang J, Hu Y, Mo L, Kim ST, Yang X, Tan A, Zhang H*, et al.* (2013) Genome-wide association study of sex hormones, gonadotropins and sex hormone-binding protein in Chinese men. *J Med Genet* 50(12):794-801.

47. Del-Aguila JL, Beitelshees AL, Cooper-Dehoff RM, Chapman AB, Gums JG, Bailey K, Gong Y, Turner ST, Johnson JA, & Boerwinkle E (2014) Genome-wide association analyses suggest NELL1 influences adverse metabolic response to HCTZ in African Americans. *Pharmacogenomics J* 14(1):35-40.

48. Prescott J, Thompson DJ, Kraft P, Chanock SJ, Audley T, Brown J, Leyland J, Folkerd E, Doody D, Hankinson SE*, et al.* (2012) Genome-wide association study of circulating estradiol, testosterone, and sex hormone-binding globulin in postmenopausal women. *PLoS One* 7(6):e37815.

49. Kiryluk K, Li Y, Scolari F, Sanna-Cherchi S, Choi M, Verbitsky M, Fasel D, Lata S, Prakash S, Shapiro S*, et al.* (2014) Discovery of new risk loci for IgA nephropathy implicates genes involved in immunity against intestinal pathogens. *Nat Genet* 46(11):1187-1196.

50. Yang C, Jie W, Yanlong Y, Xuefeng G, Aihua T, Yong G, Zheng L, Youjie Z, Haiying Z, Xue Q*, et al.* (2012) Genome-wide association study identifies TNFSF13 as a susceptibility gene for IgA in a South Chinese population in smokers. *Immunogenetics* 64(10):747-753.

51. Osman W, Okada Y, Kamatani Y, Kubo M, Matsuda K, & Nakamura Y (2012) Association of common variants in TNFRSF13B, TNFSF13, and ANXA3 with serum levels of non-albumin protein and immunoglobulin isotypes in Japanese. *PLoS One* 7(4):e32683.

52. Yu XQ, Li M, Zhang H, Low HQ, Wei X, Wang JQ, Sun LD, Sim KS, Li Y, Foo JN*, et al.* (2011) A genome-wide association study in Han Chinese identifies multiple susceptibility loci for IgA nephropathy. *Nat Genet* 44(2):178-182.

53. Yang M, Wu Y, Lu Y, Liu C, Sun J, Liao M, Qin M, Mo L, Gao Y, Lu Z*, et al.* (2012) Genome-wide scan identifies variant in TNFSF13 associated with serum IgM in a healthy Chinese male population. *PLoS One* 7(10):e47990.

54. Postmus I, Trompet S, Deshmukh HA, Barnes MR, Li X, Warren HR, Chasman DI, Zhou K, Arsenault BJ, Donnelly LA*, et al.* (2014) Pharmacogenetic meta-analysis of genome-wide association studies of LDL cholesterol response to statins. *Nat Commun* 5:5068.

55. Chu AY, Guilianini F, Grallert H, Dupuis J, Ballantyne CM, Barratt BJ, Nyberg F, Chasman DI, & Ridker PM (2012) Genome-wide association study evaluating lipoprotein-associated phospholipase A2 mass and activity at baseline and after rosuvastatin therapy. *Circ Cardiovasc Genet* 5(6):676-685.

56. Surakka I, Whitfield JB, Perola M, Visscher PM, Montgomery GW, Falchi M, Willemsen G, de Geus EJ, Magnusson PK, Christensen K*, et al.* (2012) A genome-wide association study of monozygotic twin-pairs suggests a locus related to variability of serum high-density lipoprotein cholesterol. *Twin Res Hum Genet* 15(6):691-699.

57. Inouye M, Ripatti S, Kettunen J, Lyytikainen LP, Oksala N, Laurila PP, Kangas AJ, Soininen P, Savolainen MJ, Viikari J*, et al.* (2012) Novel Loci for metabolic networks and multi-tissue expression studies reveal genes for atherosclerosis. *PLoS Genet* 8(8):e1002907.

58. Bis JC, Kavousi M, Franceschini N, Isaacs A, Abecasis GR, Schminke U, Post WS, Smith AV, Cupples LA, Markus HS*, et al.* (2011) Meta-analysis of genome-wide association studies from the CHARGE consortium identifies common variants associated with carotid intima media thickness and plaque. *Nat Genet* 43(10):940-947.

59. Smith EN, Chen W, Kahonen M, Kettunen J, Lehtimaki T, Peltonen L, Raitakari OT, Salem RM, Schork NJ, Shaw M*, et al.* (2010) Longitudinal genome-wide association of cardiovascular disease risk factors in the Bogalusa heart study. *PLoS Genet* 6(9):e1001094.
