# Supplementary material for: A phenomics-based approach for the detection and interpretation of shared genetic influences on 29 biochemical indices in southern Chinese men
Source: BMC Genomics. 2019 Dec 16;20:983. doi: 10.1186/s12864-019-6363-0 (PMC6916074; doi:10.1186/s12864-019-6363-0)
Supplement: Supplementary file 10 — Additional file 10: Table S6. The primer sequences of osteogenic and adipogenic differentiation in 3 T3-L1 cells. [file 12864_2019_6363_MOESM10_ESM.docx]

**Table S6.** The primer sequences of osteogenic and adipogenic differentiation in 3T3-L1 cells.

| gene name | Forward primer | Reverse primer |
| --- | --- | --- |
| human ALDH2 | TTATCCAGCCCACCGTGTTC | GCTGCCAGCCCATACTTAGA |
| mouse Adiponectin | TCCTGGAGAGAAGGGAGAGAAAG | CCCTTCAGCTCCTGTCATTCC |
| mouse C/EBP α | GAACAGCAACGAGTACCGGGTA | GCCATGGCCTTGACCAAGGAG |
| mouse C/EBP β | CAAGCTGAGCGACGAGTACA | CAGCTGCTCCACCTTCTTCT |
| mouse FABP4 | GATGCCTTTGTGGGAACCTG | GCCATGCCTGCCACTTTC |
| mouse qPpar γ | CAAGAATACCAAAGTGCGATCAA | GAGCTGGGTCTTTTCAGAATAATAAG |
| mouse ALP | CACCATTTTTAGTACTGGCCATCG | GCTACATTGGTGTTGAGCTTTTGG |
| mouse Osteocalcin | CTAGCAGACACCATGAGGACC | GCCGGAGTCTGTTCACTACC |
| mouse Runx2 | GACGTGCCCAGGCGTATTTC | AAGGTGGCTGGGTAGTGCATTC |
| mouse Col1 | CATGTTCAGCTTTGTGGACCTC | CCTTAGGCCATTGTGTATGCAG |
| mouse Gaphd | GTTCCTACCCCCAATGTGTC | AAGGTGGAAGAGTGGGAGTT |
